# Supplementary material for: The synergistic impact of Spirulina and selenium nanoparticles mitigates the adverse effects of heat stress on the physiology of rabbits bucks
Source: PLoS One. 2023 Jul 12;18(7):e0287644. doi: 10.1371/journal.pone.0287644 (PMC10337889; doi:10.1371/journal.pone.0287644)
Supplement: Attachment — Submitted filename: Response to Reviewers.pdf [file pone.0287644.s005.pdf]

Responses to the comments of the Editor

I found your manuscript interesting and fit the journal scope. It has also improved well after the first revision round, but there are still some revisions that need to be fixed before accepting this paper. Please make the modifications requested by the reviewers shown below.

Response:

Thank you for your time and consideration. Your comments and those of the reviewers were highly insightful and enabled us to enhance the quality of our manuscript. We have now changed this manuscript and inserted the required modifications with point-by-point responses to each of the reviewer's comments. The corrected sentences were followed using **Track Changes** in the revised manuscript.

Responses to the comments of Reviewer #4

Dear Authors, I found your MS interesting and fit the journal scope. It has also improved well after the first revision round, but there are still some revisions need to be fixed before accepting this paper.<br>**Response:** We sincerely appreciate your helpful and constructive comments. The manuscript has been improved as suggested.
Introduction: There are some important recent related references that can be added as a background to support your introduction such as:<br>- Effect of selenium nanoparticles and/or Spirulina Platensis on growth, hematobiochemical, antioxidant status, hormonal profile, immunity, and apoptosis of growing rabbits exposed to thermal stress.<br>- Impacts of selenium nanoparticles and spirulina alga to alleviate the deleterious effects of heat stress on reproductive efficiency, oxidative capacity, and immunity of doe rabbits.<br>- Zinc and/or Selenium enriched Spirulina as antioxidants in growing rabbit diets to alleviate the deleterious impacts of heat stress during summer season.<br>- Effect of rearing system and season on behavior, productive performance and carcass quality of rabbit: a review.<br>**Response:** We appreciate your suggestion and done accordingly. Kindly check (lines 73, 97, 99).
L.49: “[2-5]”: How you started your references' sequence with number 2!<br>**Response:** Thank you for the comment. Citations sequels were corrected. Kindly check (line 56).
L.58: Change “Also” to “Additionally”.<br>**Response:** Thank you for your comment and done accordingly. Kindly check (line 65).

L.60: “ROS”: Define it (expand) as it is mentioned for the first time in your text.<br>**Response:** Ok, thanks a lot for this valuable comment. ROS has been defined as reactive oxygen species (ROS). Please check (Line 71).
L.63: “8–11”: Please, add the following reference to support your statement: - Effect of bee venom on reproductive performance and immune response of male rabbits.<br>**Response:** We appreciate your suggestion and done accordingly. Kindly check (line 73).
L.72: “so”: Add “therefore,” instead.<br>**Response:** Done as suggested. Kindly check (line 82).
L.410-414: Try to go deeper in the scientific explanation. You can also refer to my suggested reference titles mentioned for the introduction to enrich your discussion as a general.<br>**Response:** We appreciate your suggestion and done accordingly. Kindly check (lines 448-455).
L.416: “[7]”: Please, add the following related references to support your current statement and your conclusion (as you mentioned "artificial insemination under heat stress"):<br>- Soliman, F. and El-Sabrout, K. (2020). Artificial insemination in rabbits: Factors that interfere in assessing its results. Journal of Animal Behaviour and Biometeorology, 8, 120–130. https://doi.org/10.31893/jabb.20016<br>- Elkomy, A.; El-Hanoun, A.; Abdella, M.; El-Sabrout, K. Improving the reproductive, immunity and health status of rabbit does using honey bee venom. J. Anim. Physiol. Anim. Nutr. 2021, 105, 975–983. https://doi.org/10.1111/jpn.13552<br>**Response:** We appreciate your suggestion and done accordingly. Kindly check (lines 466-468).

Responses to the comments of Reviewer #5

**Manuscript title:** The Synergistic Impact of Spirulina and Selenium Nanoparticles Mitigates the Adverse Effects of Heat Stress on Rabbits Bucks<br>The manuscript addresses a novel and vital topic for researchers, breeders, and others interested in the field. However, the following comments, inquiries, and suggestions may help improve the manuscript's quality.<br>**Response:** We deeply appreciate your helpful and constructive comments. The manuscript has been improved as suggested.
1. How do you judge that the conditions in the positive group are thermal-neutral?<br>**Response:** Thank you for the comment. Bucks in the 1st group (control-NC) were kept under normal conditions (11-22 ^oC; 40 - 45% RH%), while the other groups were kept under heat stress conditions (32±0.50 °C; 60-66% RH %). Kindly check (lines 29-32 and 135-138)

2. The hypothesis is not clear in the introduction. (See the comment (P:2) of the comments L:82-83).

Response: Ok, we appreciate your suggestion and was done accordingly. Kindly check (lines 102-103)

3. I recommend calculating the change caused by the combination of Spirulina (SP) and two levels of selenium nanoparticles (SP-SeNPs) in a new table.

Values that have already exceeded the average value = SP value + SP-SeNPs value/2 are placed. Keeping in mind that only the superior values are placed in the new table, and the values that are placed have a positive meaning, regardless of whether the values are + or -.

Reference should be made to the new table which you will do with the results and discussion to clarify the meaning of the synergy between the combination of SP and SP-SeNPs.

Indicate which of the two levels is the best or both according to the results in the new table and refer to this in the abstract and in the conclusion

To obtain the change % in the following

1- Calculating the average value of the (SP + SP-SeNPs).

2- Calculating the value of the change in the value when the combination of SP and SP-SeNPs in both the two levels;

The change = recorded value - (1).

3- Calculate the change as a percentage;

Change % = (2) / (1) X 100.

Table 8. The percentage change in both levels due to the synergistic impact of the SP and SP-SeNPs.

**Items**	**Average**	**Change %<br>SP+SeNPs25**	**Change %<br>SP+SeNPs50**
**Hematological variables**			
Hemoglobin	(11.93+ 10.5) /2<br>= 11.215	(12.19-11.215) /11.215*100<br>= 8.69	(12.28-11.215) /11.215*100<br>= 9.5
RBCs	6.18	2.75	2.91
…ect			
**Blood metabolites**			
…..ect			
**antioxidant and oxidative marks in the blood and seminal plasma**			
**…. Ect**			
**Semen production parameters**			
**…..**			
**Sperm variables in post-thawed**			
**…. Ect**			
**Blood and seminal testosterone**			

…..ect			
**Reproductive performance of rabbits does**			
……ect			

Response: Ok, we appreciate your suggestion and was done accordingly. Kindly check Table 8 and Lines 337-350.

Line:28, 112 ……in the 1st group Comment: a superscript letter

Response: Done as requested. Kindly check line 29.

Line 57-58: Comment: It is necessary to specify the type of change that occurred, whether by increase or decrease, rather than writing a change in general.

Response: We deeply appreciate your constructive comment. The types of changes were specified in Lines 65-67.

Line 59, 328, 369. Comment: Put the whole term in the first time in L: 59, and the abbreviation in the next. The term ROS = reactive oxygen species.

Response: Done as requested. Kindly check line 71.

Line 63……….and antiparasitic activities and contains. Comment: Remove the spaces.

Response: Done as requested. Kindly check lines 74-75.

Line 67………. animal nuitrition, and the most Comment: animal nutrition and the most.

Response: Corrected as requested. Kindly check line 79.

Line 70 . So, elements in NPs form…Comment: So, elements in the NPs form

Response: Corrected as requested. Kindly check line 82.

Line 73-74…, metabolic processes, and is an essential trace element for animals. Comment:….., and metabolic processes, and is an essential trace element for animals.

Response: Done as requested. Kindly check line 86.

Line 74….Se is a crucial component in …….Comment:… The Se is a crucial component in

Response: Corrected as requested. Kindly check line 87.

Line 82-83: SeNPs and/or Spirulina (SP) were incorporated to mitigate the negative impacts on the heat-stressed growing rabbit by improving the antioxidant status. Comment: Rewrite this sentence to be the research hypothesis.

Response: Done as requested. Kindly check lines 101-103.

Line 97……..and 3.15±0.32 kg LBW Comment: don't use the abbreviation (LBW) and use the full term "Live body weight".

Response: Done as requested. Kindly check line 120.

Line 97-99…..Each buck served as a replicate and was kept individually in stainless steel cage batteries (40×50×35cm) accommodated with feeders and automatic drinkers in a closed

system controlled by heat, humidity, and light regimes. Comment: Please remove the spaces in the previous sentence.<br>**Response:** Done as requested. Kindly check lines 121-124.
**Line 113** … under normal indoor conditions. Comment : write the (…-… °C; …-…RH%).<br>**Response:** Done as requested. Kindly check lines 135-136.
**Line 116 - 117**….Comment : What was the basis for selecting the number of hours and days that you relied on in the negative control group?<br>**Response:** We deeply appreciate your constructive comment. The basis of selection was based on a previous study (Please check line 138:<br>El-Ratel IT, Attia KAH, El-Raghi AA, Fouda SF. Relief of the negative effects of heat stress on semen quality, reproductive efficiency and oxidative capacity of rabbit bucks using different natural antioxidants. Anim Biosci. 2021;34: 844–854. doi:10.5713/ajas.20.0258
**Line 125**…. The mean PDI was 0.13, Comment : Don't use the abbreviation (PDI) and use the full term Polydispersity Index.<br>**Response:** Done as requested. Kindly check line 147.
**Line 143**…. by using a phase-contrast microscope with a hot stage at 37°C<br>Comment: Remove the spaces.<br>**Response:** Done as requested. Kindly check line 165.
**Line 161** Comment: Tab.<br>**Response:** Done as requested. Kindly check line 183.
**Line 176** ….. ear-vein of the does Comment: ear-vein of the dose<br>**Response:** Corrected as suggested. Kindly check line 199.
**Line 268: Table 3**.. .. and their combinations on oxidative bio-marks in blood and...Comment:….., and their combinations on antioxidant and oxidative marks in the blood and ….<br>**Response:** Corrected as requested. Kindly check line 290.
