# Supplementary material for: The synergistic impact of Spirulina and selenium nanoparticles mitigates the adverse effects of heat stress on the physiology of rabbits bucks
Source: PLoS One. 2023 Jul 12;18(7):e0287644. doi: 10.1371/journal.pone.0287644 (PMC10337889; doi:10.1371/journal.pone.0287644)
Supplement: Attachment — Submitted filename: reREVIEWED PONE-D-23-02023_R1 (1).pdf [file pone.0287644.s009.pdf]

PLOS ONE

The Synergistic Impact of Spirulina and Selenium Nanoparticles Mitigates the Adverse Effects of Heat Stress on Semen Quality of Rabbits Bucks

--Manuscript Draft--

**Manuscript Number:**	PONE-D-23-02023R1
**Article Type:**	Research Article
**Full Title:**	The Synergistic Impact of Spirulina and Selenium Nanoparticles Mitigates the Adverse Effects of Heat Stress on Semen Quality of Rabbits Bucks
**Short Title:**	Spirulina and Selenium Nanoparticles Mitigate Heat Stress Impacts on Rabbits Bucks
**Corresponding Author:**	Mohammed Fouad El Basuini, Professor<br>Tanta University, EGYPT<br>Tanta, Gharbeya EGYPT
**Keywords:**	Bucks; heat stress; Selenium Nanoparticles; Spirulina; Semen Quality; Sperm Fertility
**Abstract:**	Heat stress has a detrimental effect on animal fertility, particularly testicular functions, including reduced sperm output and quality, which causes an economic loss in the production of rabbits. The present trial investigated the efficacy of dietary Spirulina (SP) (Arthrospira platensis), selenium nanoparticles (SeNPs), and their combination (SP-SeNPs) on semen quality, haemato-biochemical, oxidative stress, immunity, and sperm quality of heat-stressed (HS) rabbit bucks. Sixty mature bucks (APRI line) were distributed into 6 groups of ten replicates. Bucks in the 1st group were kept under thermoneutral conditions (control-NC), while the 2nd was kept under heat stress and as a control-HS. The control groups were fed a commercial pelleted diet and the other four heat-stressed groups were fed a commercial pelleted diet with 1 g SP, 25 mg SeNPs, 1 g SP+25 mg SeNPs, and 1 g SP+50 mg SeNPs per kg diet, respectively. The dietary inclusion of SP, SeNPs, and their combinations significantly increased hemoglobin, platelets, total serum protein, high-density lipoproteins, glutathione, glutathione peroxidase, superoxide dismutase, and seminal plasma testosterone while decreased triglycerides, total cholesterol, urea, creatinine, and malondialdehyde compared with the control-HS. Red blood cells, packed cell volume, serum albumin, and testosterone significantly increased, while SeNPs, SP+SeNPs25, and SP+SeNPs50 significantly decreased low-density lipoproteins, aspartate, and alanine amino transferees. Total antioxidant capacity substantially increased in serum and seminal plasma, while seminal plasma malondialdehyde decreased in 25 or 50 mg of SeNPs+SP/kg groups. All supplements significantly improved libido, sperm livability, concentration, intact acrosome, membrane integrity, total output in fresh semen, and sperm quality in cryopreserved semen. In conclusion, the dietary inclusion of SP plus SeNPs may be a suitable tool for improving reproductive efficiency, health, oxidative stress, and immunity of bucks in the breeding strategy under hot climates.
**Order of Authors:**	Ibrahim T. El-Ratel, Ph.D.
	Mawada E. Elbasuny
	Hamdy A. El-Nagar, Professor
	Abdel-Khalek E. Abdel-Khalek, Professor
	Ali A. El-Raghi, Ph.D.
	Mohammed Fouad El Basuini, Professor
	Khaled H. El-Kholy, Ph.D.
	Sara F. Fouda, Ph.D.
**Response to Reviewers:**	Responses to the comments of the Editor<br>1- Please authors do make data available.<br>Response: Yes - all data are fully available without restriction.<br><br>2- The title of the manuscript does not express the goal of the experiment well. It is

Powered by Editorial Manager® and ProduXion Manager® from Aries Systems Corporation

suggested that the address (The Synergistic Impact of Spirulina and Selenium Nanoparticles Mitigates the Adverse Effects of Heat Stress on semen quality for Rabbits Bucks).
Response: Ok, thanks a lot for this valuable comment. The title of the manuscript has been changed. Kindly check (line 2-3).

3- The abstract needs to write a one or two line at most explaining the economic importance of this research (the negative effect of heat stress on fertility and the decline in semen quality).
Response: We appreciate your suggestion and done accordingly. Kindly check (lines 25-30). The required modifications were made as follows:
"Heat stress has a detrimental effect on animal fertility, particularly testicular functions, including reduced sperm output and quality, which causes an economic loss in the production of rabbits. The present trial investigated the efficacy of dietary Spirulina (SP) (Arthrospira platensis), selenium nanoparticles (SeNPs), and their combination (SP-SeNPs) on semen quality, haemato-biochemical, oxidative stress, immunity, and sperm fertility of heat-stressed (HS) rabbit bucks."

4- The Material and methods need write in more detail (environment, managerial and hygienic conditions for rabbits during the experiment).
Response: We deeply appreciate your comment. Kindly check (Lines 103-111 and Lines 119-126). The required modifications were made as follows:
"The study was conducted at a private commercial rabbit farm in Mansoura City, Dakahlia Governorate, Egypt, from December 2021 to April 2022. The experimental animals included 60 sexually mature APRI line bucks (7-8 months of age and 3.15±0.32 kg LBW). Each buck served as a replicate and was kept individually in stainless steel cage batteries (40×50×35cm) accommodated with feeders and automatic drinkers in a closed system with controlled heat, humidity, and light regimes (12 hrs light: 12 hrs dark) under the same managerial and hygienic conditions."

"The experimental animals (n=60) were randomly divided into six groups (10/group). Bucks in the 1st group (control-NC, G1) were kept under normal conditions of ambient temperature (11-22 oC) and relative humidity (RH) from 40 to 45% and fed on CPD without supplementation. Bucks in the 2nd group (control-HS, G2) were exposed to HS conditions (32 ± 0.50 °C; 60-66% RH) and fed on CPD without supplementation. Other four treatment groups, including bucks kept under HS conditions as in G2 and fed on CPD supplemented with 1g SP (G3), 25 mg SeNPs (G4), 1 g SP+25 mg SeNPs (G5), and 1 g SP+50 mg SeNPs (G6) per kg diet, respectively. The treatment period was two months, followed by semen collection period of 10 weeks."

5- Experimental design need write more clearly.
Response: Thank you for your comment and done accordingly. Kindly check (line 119-126).

6- The results in general need more deep discussion.
Response: Thanks a lot for this valuable comment, The results are discussed in more detail. Please check (lines 332-333, 338-339, 346-349, 405-414).

7- Clarification of the economic importance and the economic and environmental return of this experiment in all parts of the manuscript (summary, introduction, discussion of results and conclusion will strengthen the manuscript and clarify the goal of the experiment.
Response: Thanks a lot for this valuable comment, The results are discussed in more detail. Kindly check (summary line 25-27, introduction line 51-52 and discussion line 332-333, 338-339, 346-349, 405-414).

Responses to the comments of Reviewer #1
Comment: The part of Materials and methods needs more clarification about design, were the all groups reared in the same building? cages type and dimensions, type of building, the period of the year you performed the experiment, have you treated all groups (except control) to heat stress treatment?
Response: We sincerely appreciate your helpful and constructive comments. Kindly check (Lines 103-111 and Lines 119-126). The required modifications were made as follows:

Powered by Editorial Manager® and ProduXion Manager® from Aries Systems Corporation

Line 103-111:
“The study was conducted at a private commercial rabbit farm in Mansoura City, Dakahlia Governorate, Egypt, from December 2021 to April 2022. The experimental animals included 60 sexually mature APRI line bucks (7-8 months of age and 3.15±0.32 kg LBW). Each buck served as a replicate and was kept individually in stainless steel cage batteries (40×50×35cm) accommodated with feeders and automatic drinkers in a closed system with controlled heat, humidity, and light regimes (12 hrs light: 12 hrs dark) under the same managerial and hygienic conditions.”

Lines 119-126:
“The experimental animals (n=60) were randomly divided into six groups (10/group). Bucks in the 1st group (control-NC, G1) were kept under normal conditions of ambient temperature (11-22 oC) and relative humidity (RH) from 40 to 45% and fed on CPD without supplementation. Bucks in the 2nd group (control-HS, G2) were exposed to HS conditions (32 ± 0.50 °C; 60-66% RH) and fed on CPD without supplementation. Other four treatment groups, including bucks kept under HS conditions as in G2 and fed on CPD supplemented with 1g SP (G3), 25 mg SeNPs (G4), 1 g SP+25 mg SeNPs (G5), and 1 g SP+50 mg SeNPs (G6) per kg diet, respectively. The treatment period was two months, followed by semen collection period of 10 weeks.”

Responses to the comments of Reviewer #2
Comment: The manuscript represents a thorough and detailed research conducted by the researchers. Its highly commendable, though few grammatical errors were cited. If effected and published, it will stimulate readership. It also address a global challenge of mitigating adverse effects of global warming.
Response: we sincerely appreciate your effort in reviewing our manuscript and your constructive comments. The manuscript has been improved, organized, and proofread as suggested.

Responses to the comments of Reviewer #3
Comment: - Title needs modification.
Response: Ok, thanks a lot for this valuable comment. The title of the manuscript has been changed. Kindly check (line 2-3).

Comment: - It’s not clear what the authors wanted study; a) Does they wanted to study effect of spirulina on HS and nano Selenium on HS alone or in combination? b) If authors wish to study the interaction then why there is not combination supplementation in the positive control group (non-heat stress exposed group). c) How the combination will be better than the individual ones?
Response: Thank you very much for your constructive comment. Kindly check lines (27-30 and 119-126). The aim and design of the experiment has been clarified as follows:

Line 27-30:
“The present trial investigated the efficacy of dietary Spirulina (SP) (Arthrospira platensis), selenium nanoparticles (SeNPs), and their combination (SP-SeNPs) on semen quality, haemato-biochemical, oxidative stress, immunity, and sperm fertility of heat-stressed (HS) rabbit bucks.”

Lines 119-126:
“The experimental animals (n=60) were randomly divided into six groups (10/group). Bucks in the 1st group (control-NC, G1) were kept under normal conditions of ambient temperature (11-22 oC) and relative humidity (RH) from 40 to 45% and fed on CPD without supplementation. Bucks in the 2nd group (control-HS, G2) were exposed to HS conditions (32 ± 0.50 °C; 60-66% RH) and fed on CPD without supplementation. Other four treatment groups, including bucks kept under HS conditions as in G2 and fed on CPD supplemented with 1g SP (G3), 25 mg SeNPs (G4), 1 g SP+25 mg SeNPs (G5), and 1 g SP+50 mg SeNPs (G6) per kg diet, respectively. The treatment period was two months, followed by semen collection period of 10 weeks.”

Powered by Editorial Manager® and ProduXion Manager® from Aries Systems Corporation

Comment: The authors failed to highlight the need for study or impact of heat stress on the semen/reproductive performance in bucks. Authors further failed to explain why spirulina and nano Se has been selected for the study? As there is various polyphenolic compounds and nano forms of other minerals (Zn, Cr); why these nutrients or additives has been not used in the study? Why combination study is needed?
Response: Thank you for the comment. The adverse impacts of heat stress were detailed in lines 51-62. The importance of spirulina Se were written in lines 66-70. The use of SeNPs were detailed in lines 71-82. The combination studies (Spirulina + SeNPs) were in lines 86-93. Also, few studies were carried out on the effect of a combination of SP with different levels of SeNPs on the performance of male rabbits.

Comment: What is the selenium content in the CPD?
Response: Kindly check line 116.

Comment: What is the NRC recommended level of Selenium in rabbit diets?
Response: Thank you for the comment. Selenium levels in rabbit diets vary widely (0.05 - 1 mg/kg) according to dietary ingredients, rabbits' growth stage, and physiological condition.

Comment: What is the duration of supplementation or experiment period?
Response: Ok, thanks a lot for this valuable comment. The treatment period was two months followed by semen collection period of 10 weeks. Kindly check (line 126).

Comment: How semen physical quality was assessed? Was manual method or CASA was used?
Response: Thanks a lot for this valuable comment. All physical semen parameters were assessed manually.

Comment: The authors did not presented/exhibited the heat stress in bucks by exposing to cyclic HS.
Response: Thanks a lot for this valuable comment, the application of cyclic HS on semen production conflicts with the duration of spermatogenesis. So, we will need a recovery rate of about one month to study the effect of cyclic HS on sperm quality which was produced about one month before semen collection. In our study, we used a control group under normal conditions and another control group under HS conditions to assess the impaired effect of HS on different parameters studied.

Comment: No information about feed consumption since, the HS first affects the intake. No data on feed consumption, body weight change or water intake as indicator of HS induction in bucks.
Response: Thank you for your comment. Some data, such as feed consumption, body weight change or water intake, were assigned to a student graduation project and therefore were not included in the manuscript to avoid re-publishing the same data.

Comment: The results seems contradictors as HS could results in haemo-concentration then the blood parameters should be increased in negative control group compared to the TNZ or positive control. No discussion about the blood parameters were presented.
Response: Ok, thanks a lot for this valuable comment, blood parameters were presented in discussion. (Lines 401-410).

Comment: Why post-thaw semen quality was assessed? How HS is correlated with post-thaw semen quality?
Response: Ok, thanks a lot for this valuable comment. The present results revealed the effect of treatments on semen quality in fresh cases. These results are good when we use the treated bucks in natural mating. The question is whether the semen produced from each group has the ability to cryopreserve or not. So, we studied the freezing ability of semen produced from each group when used in artificial insemination.

Comment: Discussion is not clear and supplementing the findings. Needs major improvement. The authors failed to explain the synergism between the spirulina and Se.
Response: We deeply appreciate your comment. The discussion part has been

Powered by Editorial Manager® and ProduXion Manager® from Aries Systems Corporation

	improved. Kindly check lines 332-333, 338-339, 346-349, 405-414.
Additional Information:	
**Question**	**Response**
**Financial Disclosure**<br><br>Enter a financial disclosure statement that describes the sources of funding for the work included in this submission. Review the submission guidelines for detailed requirements. View published research articles from *PLOS ONE* for specific examples.<br><br>This statement is required for submission and **will appear in the published article** if the submission is accepted. Please make sure it is accurate.<br><br>**Unfunded studies**<br>Enter: *The author(s) received no specific funding for this work.*<br><br>**Funded studies**<br>Enter a statement with the following details:<br>• Initials of the authors who received each award<br>• Grant numbers awarded to each author<br>• The full name of each funder<br>• URL of each funder website<br>• Did the sponsors or funders play any role in the study design, data collection and analysis, decision to publish, or preparation of the manuscript?<br>• **NO** - Include this sentence at the end of your statement: *The funders had no role in study design, data collection and analysis, decision to publish, or preparation of the manuscript.*<br>• **YES** - Specify the role(s) played.<br><br>* typeset	The author(s) received no specific funding for this work.
**Competing Interests**<br><br>Use the instructions below to enter a competing interest statement for this submission. On behalf of all authors, disclose any competing interests that could be perceived to bias this work—acknowledging all financial support and any other relevant financial or non-financial competing interests.	The authors have declared that no competing interests exist.

This statement is required for submission and **will appear in the published article** if the submission is accepted. Please make sure it is accurate and that any funding sources listed in your Funding Information later in the submission form are also declared in your Financial Disclosure statement.<br><br>View published research articles from *PLOS ONE* for specific examples.<br><br>**NO authors have competing interests**<br><br>Enter: *The authors have declared that no competing interests exist.*<br><br>**Authors with competing interests**<br><br>Enter competing interest details beginning with this statement:<br><br>*I have read the journal's policy and the authors of this manuscript have the following competing interests: [insert competing interests here]*<br><br>* typeset	
**Ethics Statement**<br><br>Enter an ethics statement for this submission. This statement is required if the study involved:<br><br>• Human participants<br>• Human specimens or tissue<br>• Vertebrate animals or cephalopods<br>• Vertebrate embryos or tissues<br>• Field research<br><br>Write "N/A" if the submission does not require an ethics statement.<br><br>General guidance is provided below. Consult the submission guidelines for detailed instructions. **Make sure that all**	The ethical committee of the Faculty of agriculture at Tanta University approved the experimental protocol and all methods in the present study for treating animals for scientific purposes (Approval No. AY2019-2020/ Session 6/ 2020.01.13). All experiments were performed in accordance with relevant guidelines and regulations. Our reporting of research involving animals follows the recommendations of the ARRIVE guidelines.

**information entered here is included in the Methods section of the manuscript.**<br><br>**Format for specific study types**<br><br>**Human Subject Research (involving human participants and/or tissue)**<br>• Give the name of the institutional review board or ethics committee that approved the study<br>• Include the approval number and/or a statement indicating approval of this research<br>• Indicate the form of consent obtained (written/oral) or the reason that consent was not obtained (e.g. the data were analyzed anonymously)<br><br>**Animal Research (involving vertebrate animals, embryos or tissues)**<br>• Provide the name of the Institutional Animal Care and Use Committee (IACUC) or other relevant ethics board that reviewed the study protocol, and indicate whether they approved this research or granted a formal waiver of ethical approval<br>• Include an approval number if one was obtained<br>• If the study involved *non-human primates*, add *additional details* about animal welfare and steps taken to ameliorate suffering<br>• If anesthesia, euthanasia, or any kind of animal sacrifice is part of the study, include briefly which substances and/or methods were applied<br><br>**Field Research**<br><br>Include the following details if this study involves the collection of plant, animal, or other materials from a natural setting:<br>• Field permit number<br>• Name of the institution or relevant body that granted permission	
**Data Availability**<br><br>Authors are required to make all data underlying the findings described fully available, without restriction, and from the time of publication. PLOS allows rare exceptions to address legal and ethical	Yes - all data are fully available without restriction

concerns. See the PLOS Data Policy and FAQ for detailed information.<br><br>A Data Availability Statement describing where the data can be found is required at submission. Your answers to this question constitute the Data Availability Statement and **will be published in the article**, if accepted.<br><br>**Important:** Stating 'data available on request from the author' is not sufficient. If your data are only available upon request, select 'No' for the first question and explain your exceptional situation in the text box.<br><br>Do the authors confirm that all data underlying the findings described in their manuscript are fully available without restriction?	
**Describe where the data may be found in full sentences. If you are copying our sample text, replace any instances of XXX with the appropriate details.**<br><br>• If the data are **held or will be held in a public repository**, include URLs, accession numbers or DOIs. If this information will only be available after acceptance, indicate this by ticking the box below. For example: *All XXX files are available from the XXX database (accession number(s) XXX, XXX.*).<br>• If the data are all contained **within the manuscript and/or Supporting Information files**, enter the following: *All relevant data are within the manuscript and its Supporting Information files.*<br>• If neither of these applies but you are able to provide **details of access elsewhere**, with or without limitations, please do so. For example:<br><br>*Data cannot be shared publicly because of [XXX]. Data are available from the XXX Institutional Data Access / Ethics Committee (contact via XXX) for researchers who meet the criteria for access to confidential data.*	All relevant data are within the manuscript and its Supporting Information files.

The data underlying the results presented in the study are available from (include the name of the third party and contact information or URL).<br>• This text is appropriate if the data are owned by a third party and authors do not have permission to share the data.<br><br>* typeset	
Additional data availability information:	

Powered by Editorial Manager® and ProduXion Manager® from Aries Systems Corporation

Manuscript Click here to access/download;Manuscript;Manuscript.docx

Article

The Synergistic Impact of Spirulina and Selenium Nanoparticles Mitigates the Adverse Effects of Heat Stress on Semen Quality of Rabbits Bucks

Ibrahim T. El-Ratel [1], Mawada E. Elbasuny [1], Hamdy A. El-Nagar [2], Abdel-Khalek E. Abdel-Khalek [3], Ali A. El-Raghi [1], Mohammed F. M. El Basuini [4,5,] *, Khaled H. El-Kholy [1], Sara F. Fouda [6]

[1] Department of Animal, Poultry and Fish Production, Faculty of Agriculture, Damietta University, 34517, Damietta, Egypt; Ibrahimtalat@du.edu.eg , mawadaelnabawy@gamil.com , ali21384@yahoo.com , khelkholy@yahoo.com

[2] Department of Biotechnology Research, Animal Production Research Institute, Agricultural Research Center, Giza, Egypt; elnagar1970@hotmail.com

[3] Department of Animal Production, Faculty of Agriculture, Mansoura University, 35516, Mansoura, Egypt; abdelkhalk2004@yahoo.com

[4] Department of Animal Production, Faculty of Agriculture, Tanta University, 31527 Tanta, Egypt; mohamed.elbasuni@agr.tanta.edu.eg

[5] Faculty of Desert Agriculture, King Salman International University, South Sinai, Egypt; mohammed.elbasuini@ksiu.edu.eg

[6] Department of Poultry Production, Faculty of Agriculture, Mansoura University, 35516, Mansoura, Egypt; sarafikry@mans.edu.eg

***** Correspondence: m_fouad_islam@yahoo.com

Abstract: Heat stress has a detrimental effect on animal fertility, particularly testicular functions, including reduced sperm output and quality, which causes an economic loss in the production of rabbits. The present trial investigated the efficacy of dietary Spirulina (SP) (*Arthrospira platensis*), selenium nanoparticles (SeNPs), and their combination (SP-SeNPs) on semen quality, haemato-biochemical, oxidative stress, immunity, and sperm quality of heat-stressed (HS) rabbit bucks. Sixty mature bucks (APRI line) were distributed into 6 groups of ten replicates. Bucks in the 1st group were kept under thermoneutral conditions (control-NC), while the 2nd was kept under heat stress and as a control-HS. The control groups were fed a commercial pelleted diet and the other four heat-stressed groups were fed a commercial pelleted diet with 1 g SP, 25 mg SeNPs, 1 g SP+25 mg SeNPs, and 1 g SP+50 mg SeNPs per kg diet, respectively. The dietary inclusion of SP, SeNPs, and their combinations significantly increased hemoglobin, platelets, total serum protein, high-density lipoproteins, glutathione, glutathione peroxidase, superoxide dismutase, and seminal plasma testosterone while decreased triglycerides, total cholesterol, urea, creatinine, and malondialdehyde compared with the control-HS. Red blood cells, packed cell volume, serum albumin, and testosterone significantly increased, while SeNPs, SP+SeNPs25, and SP+SeNPs50 significantly decreased low-density lipoproteins, aspartate, and alanine amino transferees. Total antioxidant capacity substantially increased in serum and seminal plasma, while seminal plasma malondialdehyde decreased in 25 or 50 mg of SeNPs+SP/kg groups. All supplements significantly improved *libido*, sperm livability, concentration, intact acrosome, membrane integrity, total output in fresh semen, and sperm quality in cryopreserved semen. In conclusion, the dietary inclusion of SP plus SeNPs may be a suitable tool for improving reproductive efficiency, health, oxidative stress, and immunity of bucks in the breeding strategy under hot climates.

Keywords: Bucks; Heat Stress; Selenium Nanoparticles; Spirulina; Semen Quality; Sperm Fertility

1. Introduction

Heat stress has a detrimental effect on animal fertility and causes significant economic losses in rabbit production [2–5]. Oxidative stress performs a crucial function in semen quality, quantity, and fertility of sperm cells. Under heat stress (HS), lipid peroxidation increases, particularly when the temperature and humidity exceed the normal ranges [1,2]. HS negatively alters testicular functions and causes DNA fragmentation [3], leading to a reduction in the output and quality of spermatozoa [4] and animal well-being [5]. HS increases sperm abnormality by impairing the mitotic division of spermatocytes during spermatogenesis [1]. These negative impacts of HS on bucks are partially associated with generating free radicals [6]. Spermatozoa of rabbits are rich in metabolic activity and poly-unsaturated fatty acids in their plasma membrane; therefore, a rise in free radicals and lipid peroxidation reduces sperm fertilizing ability [2,7]. Also, HS causes many alterations in blood characteristics, antioxidant capacity, immunity, and reproduction of rabbits [8,9].

In order to lessen the harmful impacts of HS and to avoid buck infertility by limiting ROS production in rabbits, a variety of tools are available. In this way, using natural antioxidants lessens the deleterious impacts of HS on males by assisting with various physiological and metabolic processes [8–11]. Spirulina, a planktonic blue-green alga, exhibits anti-viral, anti-microbial, anti-inflammatory, hepato-protective, and antiparasitic activities and contains antioxidative pigments [12] because it contains a vibrant nutritional profile, particularly proteins, essential amino acids, fatty acids, vitamins, minerals, phytopigments, phycocyanin, polysaccharides, carotenoids, chlorophyll, and phenolic compounds [13–15].

Nano-technology is applied in animal nutrition, and the most basic approaches in this area are nanoparticles (NPs) with 1–100 nm dimensions. The NPs are characterized by higher bioavailability, specific surface area, surface center activity, catalytic and adsorption abilities, and less toxic properties [16]. So, elements in NPs form at low levels are more efficient than those in conventional forms when added to diets [17], so NPs can be easily assimilated in the digestive tract [18]. Selenium (Se) is included in several vital physiological processes, like reproductive efficiency, immune status, antioxidant defenses, stress prevention, endocrine, metabolic processes, and is an essential trace element for animals [19–21]. Se is a crucial component in the antioxidant enzyme glutathione peroxidase (GSH-Px) [22,23], which is a significant phase II detoxification enzyme to decrease the level of lipid peroxidation [9]. Dietary Se nanoparticles (SeNPs) supplementation increased the ability of absorption bioavailability, catalytic ability, and surface activity while reducing energy loss [24,25]. In rabbits, SeNPs provide functional benefits like preventive impacts against reproductive toxicity, enhancing the testosterone level, improving the quality of spermatozoa, and decreasing DNA damage of sperm [26].

SeNPs and/or Spirulina (SP) were incorporated to mitigate the negative impacts on the heat-stressed growing rabbit by improving the antioxidant status [14]. It can be used as a promoter of growth, antioxidants, stimulation of immunity, and anti-microbial agents in broilers under HS [14]. SP was reported to be an effective tool under HS conditions for improving buck's reproduction, stimulating antibodies, producing cytokines, increasing antioxidant capacity, and reducing lipid peroxidation [9,27,28]. The impacts of SP, Se, or SeNPs on growth performance are available in rabbits and broilers. However, few studies were carried out on the effect of a combination of SP with different levels of SeNPs on the performance of male rabbits. Thus, this

study aimed to explore the effectiveness of dietary Spirulina (SP) (*Arthrospira platensis*), selenium nanoparticles (SeNPs), and their combination (SP-SeNPs) on semen quality, haemato-biochemical, oxidative stress, immunity, and sperm quality of heat-stressed (HS) rabbit bucks..

2. Materials and methods

2.1. Animals and management protocol

The study was conducted at a private commercial rabbit farm in Mansoura City, Dakahlia Governorate, Egypt, from December 2021 to April 2022. The experimental animals included 60 sexually mature APRI line bucks (7-8 months of age and 3.15±0.32 kg LBW). Each buck served as a replicate and was kept individually in stainless steel cage batteries (40×50×35cm) accommodated with feeders and automatic drinkers in a closed system with controlled heat, humidity, and light regimes (12 hrs light: 12 hrs dark) under the same managerial and hygienic conditions. The feeding system of all bucks included a commercial pelleted diet (CPD) based on NRC [29]. The ingredients and chemical composition of CPD are shown in Table 1.

Table 1. Ingredients and chemical composition of the diet (CPD) fed to bucks.

Ingredient	%	Chemical analysis	
Berseem hay	30.05	Total protein %	17.75
Barley	24.60	Crude fiber %	12.38
Wheat bran	21.50	Total lipids %	2.27
Soybean meal (44% CP)	17.50	Digestible Energy Kcal/Kg diet	2500
Molasses	3.00	Ca %	1.24
Dicalcium Phosphate (DCP)	1.60	Na %	0.16
Limestone	0.95	Total phosphorus %	0.80
NaCl	0.30	Lysine %	0.98
Premix *	0.30	Methionine %	0.46
DL-Methionine	0.20	Methionine + Cystine %	0.76

* 1 kg premix contains: Biotin, 0.05 mg; Choline, 250 mg; Folic acid, 3 mg; Niacin, 50 mg; Pantothenic acid, 10 mg; Vitamin B_1, 2 mg; Vitamin B_{12}, 0.01 mg; Vitamin B_6, 2 mg; Vitamin E, 40 mg; Vitamin K_3, 2 mg; Vitamin A, 6000 IU; Vitamin B_2, 4 mg; Vitamin D_3, 900 IU; Co, 0.1 mg; Cu, 5 mg; Fe, 50 mg; I, 0.2 mg; Mn, 85 mg; Se, 0.1 mg; Zn, 50 mg.

2.2. Design of the experiment

The experimental animals (n=60) were randomly divided into six groups (10/group). Bucks in the 1st group (control-NC, G1) were kept under normal conditions of ambient temperature (11-22 ^{o}C) and relative humidity (RH) from 40 to 45% and fed on CPD without supplementation. Bucks in the 2nd group (control-HS, G2) were exposed to HS conditions (32 ± 0.50 °C; 60-66% RH) and fed on CPD without supplementation. Other four treatment groups, including bucks kept under HS conditions as in G2 and fed on CPD supplemented with 1g SP (G3), 25 mg SeNPs (G4), 1 g SP+25 mg SeNPs (G5), and 1 g SP+50 mg SeNPs (G6) per kg diet, respectively. The treatment period was two months, followed by semen collection period of 10 weeks.

Treatment of SP was in powder form (Alga Biotechnology Unit, National Research Center, Dokki, Egypt), and SeNPs treatment (Sigma-Aldrich, USA) were involved in this study. The SeNPs morphology by TEM revealed that the mean particle size of SeNPs was 70 nm and the value of ZP was −4.46 mV which showed the best sign of the potential stability of the colloidal system. The mean PDI was 0.13, indicating that nanoparticles produced by this system had the highest homogeneity in size distribution (Figure 1).

2.3. Libido and semen collection

One week before the main semen collection phase (training period), bucks were trained for the semen collection. Sexual desire (libido) was recorded, as a reaction time, as a time in seconds

that elapsed from female insertion into the male cage up to the complete ejaculation. Semen was collected by the same veterinarian in the morning (8 a.m.) one/wk. by an artificial vagina (40 - 41°C), and a teaser doe was used. During the experimental period, semen was collected for 10 successive weeks (7 wks. for fresh semen and another 3 wks. for semen cryopreservation).

2.4. Fresh semen evaluation

Semen of 70 ejaculates (10 bucks x 7 weeks) were taken and evaluated in fresh case/group, then an average of each semen parameter/rabbit was calculated. On the day of the semen collection, gel mass (if present) was removed, and net semen volume (NSV) was recorded in the graduated tube connected to the artificial vagina. The pH value was measured with a pH pen. In each semen sample, the score of mass motility (0-5) was determined; the percentage of forward sperm motility (progressive motility) was examined for about 200 sperm cells in five microscopic fields by using a phase-contrast microscope with a hot stage at 37°C (Leica DM 500, Leica Mikrosysteme Vertrieb GmbH, Wetzlar, Germany).

Percentages of sperm morphological abnormality (head and tail) and livability (live: unstained cells and dead: purple stained cells) were determined in about 200 sperm cells stained by eosin-nigrosine dual stain (5%:10%) using a light microscope at 100 × magnification. Semen was diluted with saline solution (1:99) to determine sperm cell concentration (SCC) with a Neubauer hemocytometer slide (GmbH +Co., Brandstwiete 4, 2000 Hamburg 11, Germany).

The hypo-osmotic swelling test (HOS-t) was employed to measure sperm membrane integrity by incubation (37°C for one hour) of 30 μl semen sample and 300 μl a solution with osmolarity level of 100 mOsm/kg) containing fructose (9 g) and sodium citrate (4.9 g) in 100 ml of distilled water in a water bath [6]. Responses of about 200 spermatozoa to be swollen or with

curled tails were determined, and membrane integrity was calculated. Total sperm outputs (TSO) per ejaculate was computed according to the following equations: TSO/ejaculate (10^6/ejaculate) = NSV (ml) x SCC (×106/ml). Acrosomal status, including sperm cells with intact acrosome cap, non-intact acrosome, and sperm cells without acrosome cap, were determined to calculate the percentage of intact acrosome using naphthol yellow S and erythrosine B. [2].

2.5. Semen cryopreservation

In cryopreserved semen (30 ejaculates/group) collected from 10 bucks/group during the last 3 weeks of the experiment, ejaculates without gel mass of each group were pooled, diluted (1 semen: 5 diluents) with Tris-extender. Each 100 ml of the extender contained Tris (3.028 mg), citric acid (1.675 mg), fructose (1.250 mg), gentamycin (5000 µg), 20% egg yolk, 7% glycerin, and distilled water up to 100 ml. Semen was packed in 0.25 mL straws (IVM technologies, L' Aigle, France) after the dilution process and equilibration (at 5 ◦C for 4 h), then offered at 4-5 cm overhead liquid nitrogen (LN) for 10 min, then preserved in LN at -196 ◦C for 30 days.

Cryopreserved semen straws were thawed at 37◦C for 30 s in a water bath. Post-thawed semen was evaluated for sperm forward motility, livability, abnormality, and membrane integrity as previously conducted in semen at the fresh case.

2.6. Analytical procedures

2.6.1. Blood variables

At the end of the experimental period, 5 bucks in each treatment were randomly selected for blood testing. Blood samples were gathered from the marginal ear-vein of the does after

topical anesthetized by Xylocaine 4% anesthetic into heparinized pipes to assess hematological parameters. Other blood samples were collected into non-heparinized tubes and left to clot, then centrifuged at 700 *g* for 20 min at 4°C to separate serum collection, which was stored at −20°C until subsequent analyses of blood biochemicals and antioxidant and immunity markers. Hematological parameters included hemoglobin concentration (Hb), packed-cell volume (PVC), and counts of red blood cells (RBCs), white blood cells (WBCs), and platelets using HB 7021 analyzer. Blood serum biochemical included concentrations of total protein (TP), albumin (AL), triglycerides (TG), total cholesterol (TC), high (HDL) and low-density (LDL) lipoproteins, urea, and creatinine as well as activity of aspartate (AST) and alanine (ALT) amino transferees. Globulin was determined by calculation (the differences between concentration of TP and AL. In blood serum, levels of total antioxidant capacity (TAC), glutathione (GSH), glutathione peroxidase (GPx), superoxide dismutase (SOD), and malondialdehyde (MDA) were assayed. All biochemicals, enzyme activity, and oxidative capacity studied were assayed colorimetrically by Bio-diagnostic Co. kits (www Bio-diagnostic.com) and UV-VIS Auto spectrophotometer (UV-2602, Labomed, Los Angeles, CA, USA). Also, enzyme-immunoassay of serum testosterone profile was employed using commercial kits (Biosource-Europe S.A. 8, rue de L'Lndustrie. B-1400 Nivelles, Belgium). Coefficients of intra- and inter-assay were 7.8 and 8.4%, respectively. The minimum and maximum detectable limits were 0.1 and 18.0 ng/ml, respectively.

2.6.2. Seminal plasma variables

Samples of fresh semen collected at the last week of the semen collection (the 7th week) from 10 bucks per group were centrifuged (700 *g* for 20 min) to obtain the seminal plasma, which was stored at –20°C) for determining TAC, MDA, and testosterone. Protein carbonyl levels (POC) in sperm media of the post-thawed semen collected during the last semen collection

period (the 10th week) were also determined. POC was determined with MBS2600784 protein carbonyl ELISA kit (MyBioSource, Giza, Egypt) based on the manufacturer's technique [30].

2.6.3. Evaluation of sperm fertility in bucks

Sperm fertility of fresh semen of each group was performed by using 120 sexually mature APRI rabbit females. All does were divided into 6 groups (n = 20), then does in each group naturally mated with 5 bucks/treatment (4 does/buck) during 3 days as a mating interval at the end of the experiment. On days 10-12 post-mating, manual abdominal palpation was performed to determine the pregnancy rate (PR). PR = (positive does (n)/mated does (n) ×100. After parturition, the kindling rate (KR) as numbers of kindled does divided by the number of positive does ×100 was calculated. Also, the litter size of does and viability rate of kits (total born and alive) were calculated. At weaning (28 days of kit age), litter size and viability rates (live and dead) were recorded.

2.7. Statistical analysis

Statistical analysis comprised the General Linear Model procedures using SAS (Cary, NC, USA: SAS Institute Inc, 2012) to study the effect of treatment by the GLM model: $Y_{ij} = \mu + T_i + e_{ij}$, where Y_{ij}, observations; μ, overall mean; T_i, effect of treatments (i, 1-6); e_{ij}, random error. Tukey's Studentized Range test was used to separate the differences between treatment groups at $P < 0.05$.

3. Results

3.1. Blood hematology and serum biochemistry

Data in Table 2 illustrate the impacts of SP, SeNPs, and their combinations on blood hematology and serum biochemistry. Significant alterations ($p < 0.01$) in all blood hematology parameters were observed except WBCs ($p=0.1538$). In comparing control-NC and control-HS groups, the results indicated the harmful effects of HS on all hematological and biochemical variables studied. In comparison with the control-HS group, all treatments increased Hb concentration and platelet count ($P<0.05$). The counts of RBCs and PCV were increased in SeNPs, SP+SeNPs25, and SP+SeNPs50 groups.

All serum biochemical parameters were statistically affected ($p<0.001$) by the dietary SeNPs enriched with SP (Table 2). All treatments substantially ($p<0.05$) boosted serum total protein and HDL, while significantly ($P<0.05$) decreased TG, TC, urea, and creatinine concentration as compared to the control-HS. The concentration of serum AL increased ($P<0.0$), while LDL, AST, and ALT decreased ($P<0.05$) in SeNPs, SP+SeNPs25, and SP+SeNPs50 as compared to the control-HS and SP groups. The concentration of serum GL significantly ($P<0.05$) increased in the SP+SeNPs50 group compared to other groups. The control-HS group exhibited the poorest values of all parameters studied above compared to the treated and control-NC groups.

Table 2. Effect of dietary supplemental Spirulina, selenium nanoparticles, and their combinations on hematological and biochemical parameters of rabbit bucks under heat stress conditions.

Items	Control-NC	Treatments (mg/kg diet)					
		**Control-HS**	**SP**	**SeNPs**	**SP+SeNPs25**	**SP+SeNPs50**	***P*-value**
**Hematological variables**							
Hemoglobin (g/dl)	12.5±0.08^{a}	9.18±0.05^{d}	11.93±0.04^{b}	10.5 ±0.14^{c}	12.19 ±0.09^{ab}	12.28± 0.03^{ab}	<0.001
Red blood cells (10^6/mm^3)	6.81±0.09^{a}	5.88± 0.2^{c}	6.10 ±0.06^{bc}	6.26±0.03^{b}	6.35± 0.03^{b}	6.36 ±0.02^{b}	<0.001
White blood cells (10^3/mm^3)	7.38±0.05	7.08 ± 0.05	7.19± 0.02	7.26± 0.03	7.25± 0.02	7.19± 0.02	0.1538
Platelets (10^3/mm^3)	253.1±1.5^{a}	205.02±3.1^{c}	219.24± 3.1^{b}	221.12±3.7^{b}	226.7±1.01^{b}	224.23± 2.2^{b}	<.0001
Packed-cell volume (%)	39.6±1.1^{a}	27.8± 1.5^{d}	30.3±0.93^{cd}	32.9±1.02^{bc}	34.6±0.7^{b}	33.48± 1.4^{bc}	<.0001
**Blood metabolites**							
TP (g/dl)	7.02±0.06^{a}	6.13 ±0.08^{c}	6.28 ±0.03^{b}	6.31± 0.04^{b}	6.40 ± 0.02^{b}	6.41± 0.02^{b}	<0.001
Albumin (g/dl)	4.01±0.034^{a}	3.21±0.015^{c}	3.25±0.02^{bc}	3.26± 0.024^{b}	3.31±0.023^{b}	3.30±0.01^{b}	<0.001
Globulin (g/dl)	3.02±0.09^{ab}	2.92± 0.08^{b}	3.03 ±0.02^{ab}	3.04 ± 0.03^{ab}	3.09 ±0.03^{ab}	3.11± 0.03^{a}	<0.001
Triglycerides (mg/dl)	56.61±1.36^{d}	86.24±1.711^{a}	78.27± 1.15^{b}	79.42 ±0.83^{b}	66.68 ±0.54^{c}	67.95±0.78^{c}	<0.001
Total cholesterol (mg/dl)	83.08±1.6^{c}	114.29 ± 1.8^{a}	97.50±1.7^{b}	96.83± 3.3^{b}	81.98±2.1^{c}	82.95±2.3^{c}	<0.001
High-density lipoproteins (mg/dl)	35.67±1.4^{a}	22.17 ± 1.1^{d}	26.23± 1.3^{c}	28.88± 1.3^{cb}	31.86± 1.3^{ab}	32.13±1.6^{ab}	<0.001
Low-density lipoproteins (mg/dl)	38.95 ±1.1^{b}	50.47 ± 2.3^{a}	47.94 ±2.4^{a}	42.35±1.3^{b}	41.42±1.3^{b}	40.16±1.4^{b}	0.0004
Aspartate amino transferase (IU)	23.65±1.1^{c}	34.32±1.3^{a}	30.23±1.6^{ab}	29.27 ± 1.3^{b}	26.66±1.8^{bc}	27.69±1.4^{bc}	0.0007
Alanine aminotransferase (IU)	20.05±1.8^{c}	28.09±1.3^{a}	26.48 ±0.9^{ab}	23.46 ± 1.4^{bc}	22.73 ±0.95^{cb}	23.15±0.75^{bc}	0.0026
Urea (mg/dl)	28.04±1.5^{c}	39.58 ± 1.9^{a}	34.48±1.2^{b}	34.04 ±1.7^{b}	31.75 ±1.9^{bc}	31.05 ±1.6^{bc}	0.0012
Creatinine (mg/dl)	1.06±0.023^{d}	1.41±0.047^{a}	1.26 ±0.025^{b}	1.18±0.027^{bc}	1.19±0.041^{bc}	1.15±0.042^{cd}	<.0001

Control-NC: control under normal conditions, Control-HS: control under heat stress conditions; SP: spirulina; SeNPs: selenium nanoparticles; SP+SeNPs25: 1g SP + 25 mg SeNPs; SP+SeNPs50: 1g SP+50 mg SeNPs

Values with distinctive superscripts within a row are varied ($P < 0.05$)

3.2. Oxidative status in serum and seminal plasma

The impacts of the dietary Spirulina, selenium nanoparticles, and their interaction on redox status in serum are revealed in Table 3. Antioxidant capacity markers were considerably ($P<0.05$) lower in the control-HS group than in the control-NC group, indicating the effects of HS on increasing lipid peroxidation in serum and seminal plasma. All dietary supplementations to buck diets increased ($P<0.05$) GSH, GPx, and SOD in serum, while decreasing ($P<0.05$) MDA level as compared to the control-HS. Enriching rabbit diets with 25 or 50 mg of SeNPs plus SP/kg diet produced significantly ($P<0.05$) the highest levels of TAC compared to other groups. Concerning the antioxidant activities in the seminal plasma of bucks, TAC increased ($p < 0.05$). At the same time, the MDA level decreased ($P<0.05$) by feeding rabbit diets with 25 or 50 mg of SeNPs plus SP/kg compared to other groups. The control-HS group exhibited the lowest activities of all the above antioxidant markers compared to the treated and control-NC.

3.3. Libido and semen production

The libido of rabbit bucks was improved ($p<0.05$) by the dietary treatments compared to the control-HS group but did not differ from that of the control-NC. Mass motility score and progressive motility percentage significantly ($P<0.05$) increased by SeNPs or their combination with SP compared to the control-HS and SP groups. All supplements significantly ($P<0.05$) increased sperm livability, SCC, intact acrosome, membrane integrity, and TSO while decreasing abnormality compared with the control-HS group. The differences in NSV among the experimental groups were not significant. The most improvement in semen characteristics was by SP+SeNPs50, but still considerably ($P<0.05$) lower than the control-NC, except RT and mass motility (Table 4).

Table 3. Effect of dietary supplemental Spirulina, selenium nanoparticles and their combinations on oxidative bio-marks in blood and seminal plasma of rabbit bucks under heat stress conditions.

Items	Control-NC	Treatments (mg/kg diet)					
		Control-HS	SP	SeNPs	SP+SeNPs25	SP+SeNPs50	***P*-value**
**Antioxidant and oxidative markersin the blood**							
Total antioxidant capacity (nmol/L)	1.94 ± 0.03^{a}	0.86 ±0.04^{c}	0.95±0.02^{bc}	0.98± 0.05^{bc}	1.22± 0.08^{b}	1.36± 0.08^{b}	<.0001
glutathione (mg/dl)	19.5±0.8^{a}	9.3± 0.5^{d}	12 ± 0.4^{c}	13.34 ±0.4^{c}	15.3±0.8^{b}	15.3±0.5^{b}	<.0001
glutathione peroxidase (IU)	3.53 ± 0.14^{a}	1.42±0.08^{d}	2.06± 0.08^{c}	2.28± 0.09^{bc}	2.37± 0.03^{b}	2.44± 0.04^{b}	<.0001
superoxide dismutase (IU)	32.3± 1.04^{a}	19.4± 0.8^{c}	22.8± 0.6^{b}	23.6± 1.6^{b}	23.8±0.5^{b}	24.5± 0.8^{b}	<.0001
malondialdehyde (nmol/mL)	4.29± 0.3^{c}	8.13±0.5^{a}	6.24± 0.1^{b}	6.22± 0.1^{b}	6.17± 0.1^{b}	6.14± 0.05^{b}	<.0001
**Antioxidant and oxidative markersin the seminal plasma**							
Total antioxidant capacity (nmol/L)	22.65± 1.3^{a}	10.01± 1.8^{c}	10.62±1.02^{c}	12.85±0.68^{bc}	15.1±0.82^{b}	15.24±0.71^{b}	<.0001
malondialdehyde (nmol/mL)	35.06±2.9^{b}	51.88±1.9^{a}	50.56±1.7^{a}	46.4± 1.2^{ab}	45.91± 1.5^{b}	45.8± 1.05^{b}	0.0002

Control-NC: control under normal conditions, Control-HS: control under heat stress conditions; SP: spirulina; SeNPs: selenium nanoparticles; SP+SeNPs25: 1g SP + 25 mg SeNPs; SP+SeNPs50: 1g SP+50 mg SeNPs. Values with distinctive superscripts within a row are varied ($P < 0.05$**)**

Table 4. Effect of dietary supplemental Spirulina, selenium nanoparticles and their combinations on semen quality parameters of rabbit bucks under heat stress conditions.

Items	Control-NC	Treatments (mg/kg diet)					
		**Control-HS**	**SP**	**SeNPs**	**SP+SeNPs25**	**SP+SeNPs50**	***P*-value**
Reaction time (sec.)	14.0 ±0.7^{c}	23.7 ± 0.6^{a}	20.8±1.3^{b}	19.2±1.1^{b}	14.3±0.7^{c}	13.9±0.6^{c}	<.0001
Net semen volume (ml)	0.75 ±0.05	0.55±0.02	0.66±0.02	0.64±0.03	0.65±0.03	0.67±0.02	0.069
Mass motility	3.8±0.2^{a}	2.6±0.16^{c}	2.9±0.2^{bc}	3.3±0.15^{ab}	3.5±0.17^{a}	3.5±0.17^{a}	0.0002
Progressive motility (%)	82.5±2.0^{a}	55 ± 2.6^{d}	75±3.4^{cd}	63.5±2.6^{bc}	66.5±3.3^{b}	70.5± 2.3^{b}	<.0001
Sperm livability (%)	85.9±1.8^{a}	51.1±1.6^{e}	63.4±1.03^{d}	68.5± 1.7^{c}	71.7±1.17^{bc}	72.8±0.8^{b}	<.0001
Sperm abnormality (%)	11.8 ±0.9^{c}	24.9±0.9^{a}	21.2±1.05^{b}	20.8±1.05^{b}	20.4± 1.7^{b}	18.2±0.9^{b}	<.0001
Sperm cell concentration (10^6/ml)	356.1±2.2^{a}	196.5±5.7^{f}	224.8± 3.2^{e}	254.3±3.5^{d}	285.5±3.9^{c}	296.8±2.6^{b}	<.0001
Intact acrosome (%)	89.4 ±1.6^{a}	60.9±1.04^{e}	74.8± 1.2^{d}	73.7±0.9^{d}	78.2± 0.8^{c}	83.8±0.9^{b}	<.0001
Membrane integrity (%)	45.1 ±1.4^{a}	21.3 ±0.7^{d}	26.1±0.9^{c}	26.7± 0.6^{c}	36.7± 1.04^{b}	37.1±0.8^{b}	<.0001
Total Sperm output (10^6/ejaculate)	266.6±17.9^{a}	107.4±4.9^{e}	148.7±4.3^{d}	162.8± 6.1^{cd}	185.1±9.1^{bc}	200.09±6.7^{b}	<.0001

Control-NC: control under normal conditions, Control-HS: control under heat stress conditions; SP: spirulina; SeNPs: selenium nanoparticles; SP+SeNPs25: 1g SP + 25 mg SeNPs; SP+SeNPs50: 1g SP+50 mg SeNPs. Values with distinctive superscripts within a row are varied ($P < 0.05$)

3.4. Sperm Quality in Post-Thawed Semen

The effects of dietary treatment supplements on sperm properties in post-thawed semen are presented in Table 5. The present results indicated that the dietary SP, SeNPs, and their combination supplements significantly (*P* <0.05) enhanced sperm quality in cryopreserved semen in terms of increasing SPM, SL, and MI and decreased sperm abnormality and PCO in comparison with the control-HS. It is of interest to note the maximum improvement occurred in sperm characteristics after thawing by SP+SeNPs50 as compared to the control-HS still to be lower than that exhibited in the control-NC group.

3.5. Testosterone in serum and seminal plasma

Results illustrated in Table 6 showed that testosterone level was significantly (*P* <0.05) increased by SeNPs and their combinations in blood serum with SP, while significantly (*P* <0.05) increased in the seminal plasma by all supplements.

3.6. Sperm fertility

The dietary treatment of bucks used in natural mating of does with both SP plus SeNPs significantly (p<0.05) increased pregnancy rate (PR) and delivered rate (DR) as compared to the control-HS other treatments, but PR and DR were similar to that in the control-NC group. SeNPs and their combinations with SP significantly (P<0.05) improved litter size at birth (Total and live) and weaning, and viability rate only at birth (Table 7).

Table 5. Effect of dietary supplemental Spirulina, selenium nanoparticles and their combinations on sperm variables in post-thawed heat-stressed rabbit semen.

Items	Control-NC	Treatments (mg/kg diet)					
		Control-HS	SP	SeNPs	SP+SeNPs25	SP+SeNPs50	*P*-value
Progressive motility (%)	72.4± 1.2^{a}	35.1±0.8^{e}	41.2±1.1^{d}	55.5±1.5^{c}	56.4±1.5^{c}	60.9±1.4^{b}	<.0001
Sperm livability (%)	68.9±1.4^{a}	42.3±0.9^{d}	54.6±0.6^{c}	54.6±1.4^{c}	61.5±0.8^{b}	62.8±0.8^{b}	<.0001
Sperm abnormality (%)	13.1±0.7^{d}	26.9± 0.8^{a}	24.5± 0.7^{b}	22.1±0.9^{b}	21.8±1.1^{cb}	21.5±1.2^{c}	<.0001
Membrane integrity (%)	73.5±0.8^{a}	40.7±0.9^{e}	47.4±1.03^{d}	56.8±1.2^{c}	58.2±1.1^{c}	62.30±1.09^{b}	<.0001
protein carbonyl (ng/mg)	2.38± 0.04^{c}	4.04±0.06^{a}	3.80± 0.05^{b}	3.66± 0.12^{b}	3.63± 0.06^{b}	3.58 ± 0.08^{b}	<.0001

Control-NC: control under normal conditions, Control-HS: control under heat stress conditions; SP: spirulina; SeNPs: selenium nanoparticles; SP+SeNPs25: 1g SP + 25 mg SeNPs; SP+SeNPs50: 1g SP+50 mg SeNPs. Values with distinctive superscripts within a row are varied ($P < 0.05$**)**

Table 6. Effect of dietary supplemental Spirulina, selenium nanoparticles and their combinations on blood and seminal testosterone of rabbit bucks under heat stress conditions.

Testosterone (ng/ml)	Control-NC	Treatments (mg/kg diet)					
		Control-HS	SP	SeNPs	SP+SeNPs25	SP+SeNPs50	*P*-value
Serum	3.65±0.053^{a}	2.09 ± 0.029^{c}	2.15±0.052^{c}	2.26±0.019^{b}	2.26±0.016^{b}	2.29±0.017 ^{b}	<.0001
Seminal Plasma	3.18±0.033^{a}	2.02±0.067^{d}	2.05±0.057^{cd}	2.17±0.024^{bc}	2.21± 0.019 ^{b}	2.26±0.024^{b}	<.0001

Control-NC: control under normal conditions, Control-HS: control under heat stress conditions; SP: spirulina; SeNPs: selenium nanoparticles; SP+SeNPs25: 1g SP + 25 mg SeNPs; SP+SeNPs50: 1g SP+50 mg SeNPs. Values with distinctive superscripts within a row are varied (*P* < 0.05**).**

Table 7. Reproductive performance of rabbits does mate by rabbit bucks treated Spirulina, selenium nanoparticles, and their combinations under heat stress conditions.

Items	Control-NC	Treatments (mg/kg diet)					
		**Control-HS**	**SP**	**SeNPs**	**SP+SeNPs25**	**SP+SeNPs50**	***P*-value**
Total mated rabbit does (n)	30	30	30	30	30	30	--
Pregnant rabbit does (n, %)	27/30 (90)^{a}	17 (56.67)^{b}	19 (63.33)^{b}	19 (63.33)^{b}	25 (83.33)^{a}	26 (86.67)^{a}	--
Delivered rabbit does (n, %)	27/27 (100)^{a}	13 (76.47)^{b}	15 (78.94)^{b}	16 (84.21)^{ab}	23 (92.00)^{a}	25 (96.15)^{a}	--
Total litter size at birth (n)	9.04 ±0.37^{a}	5.54±0.38^{e}	5.87± 0.27^{de}	6.81±0.41^{cd}	7.65±0.27^{bc}	8.04±0.23^{b}	<.0001
Live litter size at birth (n)	8.7±0.3^{a}	4.3±0.31^{d}	5.07±0.35^{d}	6.0± 0.4^{c}	6.96± 0.24^{b}	7.44± 0.24^{b}	<.0001
Viability rate at birth (%)	96.7± 1.1^{a}	78.8± 4.6^{c}	85.44±3.5^{bc}	88.5± 2.6^{b}	91.4± 1.8^{ab}	92.68±1.9^{ab}	--
Litter size at weaning (n)	8.33 ±0.33^{a}	3.92± 0.33^{d}	4.67± 0.29^{d}	5.63± 0.33^{c}	6.65± 0.25^{b}	7.12± 0.21^{b}	<.0001
Viability rate at weaning (%)	95.80± 1.3	91.79±3.05	93.75± 2.86	94.916± 2.16	95.70 ± 1.42	96.21± 1.30	--

Control-NC: control under normal conditions, Control-HS: control under heat stress conditions; SP: spirulina; SeNPs: selenium nanoparticles; SP+SeNPs25: 1g SP + 25 mg SeNPs; SP+SeNPs50: 1g SP+50 mg SeNPs. Values with distinctive superscripts within a row are varied (*P* < 0.05**).**

4. Discussion

Heat stress has an undesirable results on the health and fertility of animals and leads to substantial economic losses in the production of rabbits [2-5]. In subtropical regions, the harmful effects of HS on livestock are not easy to mitigate, especially when the high ambient temperature is combined with high humidity. In the present study, the temperature–humidity index (THI) indicated that rabbits in treatment and control-HS groups are exposed to severe heat stress (28.9 to <30.0) [31]. The positive effects of SP or Se in diets of rabbits or broilers under HS conditions, and dietary SP supplementation, as a functional component, due to its high bio-active components and its functional properties have previously been reported [32,33]. To our knowledge, the present study is the 1st to illustrate the cooperation of SP with different levels of SeNPs on the semen characteristics of heat-stressed bucks.

In the current study, the dramatic reduction observed in the quality and function of buck spermatozoa by HS was reported by El-Ratel et al. [34]. HS condition inhibits hypothalamic gonadotropin-releasing hormone (GnRH) synthesis and secretion. The function of the testis, testosterone production, and the quality of rabbit semen were mainly affected by GnRH [35]. As proved in our study, HS negatively influences the motility and membrane integrity of bucks in the control-HS compared with the control-NC group due to the ROS generation in the testicular tissues of bucks, leading to lipid peroxidation (antioxidant defense system damage) in the sperm plasma membrane [36]. The obtained results indicated the deleterious impact of HS on reducing testosterone secretion in bucks of control-HS group via a reduction in testosterone production as a result of destroying the function of Leydig cells [37]. The RT is controlled by the hypothalamus–pituitary–testis axis, nervous system, hormones, and sexual pheromones [38]. Therefore, enhancing testosterone biosynthesis [3,27] improved the sexual desire of bucks in all

treatment groups by decreasing RT compared with the control-HS group. Testosterone is required for spermatogenesis and maturation of sperm cells [39], leading to a marked reduction in abnormality and acrosomal damage. In association with enhancing the testosterone profile, antioxidant administration (SP, SeNPs, or their combinations) improved, quantitatively and qualitatively, the semen production, including mass motility score, and the percentages of sperm progressive motility, livability, normality, acrosome intact and integrity of membrane, SCC, and TSO which adversely affected by HS in rabbits [2,3]. The pronounced improvement in semen characteristics in our study was by SP+SeNPs50, but still lower ($P<0.05$) than those of bucks in the control-NC, except RT and mass motility score. In this context, a water supplementation of SeNPs significantly improved semen characteristics in rabbit bucks [24]. Se supplementations had beneficial actions via increasing serum testosterone concentrations, enhanced semen quality, and reduced sperm DNA damage in rabbit bucks [40]. These improvements may be due to the structure and bioavailability of SeNPs molecules versus the inorganic form of Se to cover the needs of the testis and epididymal segments from Se required for spermatogenesis, consequently, high-fertile sperm production and high-quality semen [24]. Supplementation of organic Se also enhanced the quality of semen and seminal plasma of heat-stressed rabbit bucks by improving the accessory sex gland functions [3]. Also, the motility, livability, and SCC of sperm were enhanced by Se treatments [41,42].

As a result, we addressed the positive impacts on fresh semen quality of bucks treated with SeNPs enriched with SP combinations, but the main question 'Are bucks with these treatments, particularly during hot climates, have profits on sperm freezing and fertilizing capabilities after cryopreservation? The outcomes in this context showed that rabbit bucks with different levels of SeNP+SP combinations substantially improved sperm characteristics in post-

thawed semen. This improvement concerns good quality semen produced by bucks in treatment groups. In rabbits, sperm characteristics in cryopreserved semen are associated with their characteristics before cryopreservation.

Antioxidant enzymes are reduced when bucks are exposed to HS [43]. Increasing MDA level causes organ damage in rabbits [44] in association with lower reproductive performance of bucks [2]. The harmful effects of HS on antioxidant enzymes and MDA levels were observed for bucks in the control-HS in comparison with the control-NC group. Therefore, decreasing oxidative stress is a crucial means of mitigating heat-stressed rabbits. The enhancement in the semen quality of bucks in treated groups in the current study was mainly related to the properties of either SP [15] or Se [3] alone and/or their synergetic effects, as antioxidants, which protect the cells from damage by enhancing antioxidant enzyme (GSH, GPx, and SOD) activity as well as reducing MDA level in serum and seminal plasma. The antioxidants scavenge ROS in the testes to decrease the endogenous oxidative damage by increasing antioxidant enzyme activities [45]. The antioxidant enzyme, SOD, converts superoxide anions to H_2O_2, which GPx catalyzes to H_2O or alcohol [46]. The reduction in MDA production by SP and SeNPs combinations mitigated the oxidative stress condition. SP contains β-carotene, Se, polypeptides, pigments, tocopherol, and phenols. Phycocyanin, the primary pigment in SP, plays a vital role in potent antioxidant effects [47,48]. In this context, both enzymatic and non-enzymatic antioxidant defense mechanisms were affected positively by Se addition in the diet of poultry [46].

It is worth noting that SeNPs enriched with SP at levels of 0.25 and 50 mg/kg diet improved the hematological blood parameters. A hematological and biochemical blood parameter represents the animal's health [49]. Considering the findings of our study, SeNPs enriched with SP showed a significant increase in Hb, RBCs, PLT, and PCV compared to the

control-HS group because the dietary photogenic, as an immune-modulatory agent, produced a significantly higher erythrogram than a control group in heat-stressed rabbits [20,50,51]. This improvement may indicate that treating SeNPs enriched with SP can protect rabbits from anemia under HS conditions [52].

Our results indicated deleterious effects of HS on all hematological parameters studied in terms of decreasing Hb concentration, RBCs and platelets count, and PCV ($P<0.05$) in control-Hs as compared to control-NC. It is worth noting that SeNPs enriched with SP at levels of 0.25 and 50 mg/kg diet improved the hematological blood parameters. A hematological and biochemical blood parameter represents the animal's health [49]. Considering the findings of our study, SeNPs enriched with SP showed a significant increase in Hb, RBCs, PLT, and PCV compared to the control-HS group because the dietary photogenic, as an immune-modulatory agent, produced a significantly higher erythrogram than a control group in heat-stressed rabbits [20,50,51]. This improvement may indicate that treating SeNPs enriched with SP can protect rabbits from anemia under HS conditions [52].

In our study, HS reduced total protein and increased TC concentrations in the serum of bucks in the control-HS, which agrees with the results of Liang et al. [9], who found a decrease in total protein and an increase in TC in heat-stressed rabbits. HS condition elevates the secretion of glucocorticoid, which stimulates gluconeogenesis [53]. The observed improvement of serum total protein and its main components (albumin and globulin) in association with reducing urea and creatinine levels in the serum of bucks in treatment groups compared to the control-HS may indicate improving protein utilization of treated bucks under HS. Also, the contents of proteins, essential amino acids, minerals, vitamins, phospholipids, and antioxidants in SP may cause improvement in protein utilization [13,54]. The hypolipidemic activities of SP, SeNPs, or their

combinations in reducing TC and TG, and increasing HDL concentration in serum were reported in animals and chickens [55,56]. Such effect may be attributed to the fact that all supplements could decrease absorbed and synthesized cholesterol in the poultry gut [50]. In the same context, the polyphenolic contents in SP could inhibit pancreatic lipase activity and decrease the lipids in blood [57].

The results showed that AST and ALT activities increased in bucks exposed to HS in the control-HS. Similarly, Maria et al. [43] reported that HS showed damage and inflammation in the liver. According to the present results in our study, reducing AST and ALT activity as well as urea and creatinine in the serum of bucks in treatment groups may suggest a safe use of SP, SeNPs, or their combination on the normal liver and kidney functions of bucks.

In rabbits, semen variables are the significant factors affecting the reproductive performance of doe rabbits [7]. Increasing the percentages of motility and normality, as well as concentrations of spermatozoa, is associated with an enhancement in fertility [2]. Additionally, sperm motility percentage affected the number of kits of doe rabbits. In mammals, sperm characteristics are essential in determining sperm fertility. The present study showed higher sperm fertility in a bucks-fed diet supplemented with SP+SeNPs combinations due to the protection of spermatozoa from lipid peroxidation, which decrease the injury and damage of sperm cells, resulting in an enhancement in male fertility [3,45,58].

Conclusion

Dietary inclusion of *spirulina platensis*, selenium nanoparticles, or their combinations (SP-SeNPs) could be potentially used as potent antioxidants for improving the reproductive performance of rabbit bucks by eliminating the deleterious effects of the oxidative stress under

heat stress conditions via enhancing sexual desire, semen characteristics, fertilizing ability, health and antioxidant statuses, and immunity of rabbit bucks. The combinations of Spirulina (1 g) plus each level of SeNPs (25 or 50 mg) supplemented to the diet provides the most benefits, which can be recommended as a valuable tool for farming rabbit bucks utilized in natural mating or artificial insemination under heat stress circumstances.

References

1. Attia YA, El-Naggar AS, Abou-Shehema BM, Abdella AA. Effect of Supplementation with Trimethylglycine (Betaine) and/or Vitamins on Semen Quality, Fertility, Antioxidant Status, DNA Repair and Welfare of Roosters Exposed to Chronic Heat Stress. Animals. 2019;9: 547. doi:10.3390/ani9080547
2. El-Ratel IT, Attia KAH, El-Raghi AA, Fouda SF. Relief of the negative effects of heat stress on semen quality, reproductive efficiency and oxidative capacity of rabbit bucks using different natural antioxidants. Anim Biosci. 2021;34: 844–854. doi:10.5713/ajas.20.0258
3. Hosny NS, Hashem NM, Morsy AS, Abo-elezz ZR. Effects of Organic Selenium on the Physiological Response, Blood Metabolites, Redox Status, Semen Quality, and Fertility of Rabbit Bucks Kept Under Natural Heat Stress Conditions. Front Vet Sci. 2020;7. doi:10.3389/fvets.2020.00290
4. Türk G, Çeribaşı AO, Şimşek ÜG, Çeribaşı S, Güvenç M, Özer Kaya Ş, et al. Dietary rosemary oil alleviates heat stress-induced structural and functional damage through lipid peroxidation in the testes of growing Japanese quail. Anim Reprod Sci. 2016;164: 133–143. doi:10.1016/j.anireprosci.2015.11.021

5. Ayo JO, Obidi JA, Rekwot PI. Effects of Heat Stress on the Well-Being, Fertility, and Hatchability of Chickens in the Northern Guinea Savannah Zone of Nigeria: A Review. ISRN Vet Sci. 2011;2011: 1–10. doi:10.5402/2011/838606
6. El-Desoky NI, Hashem NM, Elkomy A, Abo-elezz ZR. Physiological response and semen quality of rabbit bucks supplemented with Moringa leaves ethanolic extract during summer season. Animal. 2017;11: 1549–1557. doi:10.1017/S1751731117000088
7. Attia YA, Hamed RS, Bovera F, Abd El-Hamid AE-HE, Al-Harthi MA, Shahba HA. Semen quality, antioxidant status and reproductive performance of rabbits bucks fed milk thistle seeds and rosemary leaves. Anim Reprod Sci. 2017;184: 178–186. doi:10.1016/j.anireprosci.2017.07.014
8. Kholy K, Wafa W, Nagar H, Aboelmagd A, Ratel I. Physiological response, testicular function, and health indices of rabbit males fed diets containing phytochemicals extract under heat stress conditions. J Adv Vet Anim Res. 2021;8: 256. doi:10.5455/javar.2021.h510
9. Liang Z-L, Chen F, Park S, Balasubramanian B, Liu W-C. Impacts of Heat Stress on Rabbit Immune Function, Endocrine, Blood Biochemical Changes, Antioxidant Capacity and Production Performance, and the Potential Mitigation Strategies of Nutritional Intervention. Front Vet Sci. 2022;9: 906084. doi:10.3389/fvets.2022.906084
10. Abdel-Hady SB. Effect of dietary protein levels on the productive and reproductive performance of rabbits., 11: 167-177. Egypt J Rabbit Sci. 2001;11: 167–177.
11. Sheiha AM, Abdelnour SA, Abd El-Hack ME, Khafaga AF, Metwally KA, Ajarem JS, et al. Effects of Dietary Biological or Chemical-Synthesized Nano-Selenium Supplementation on Growing Rabbits Exposed to Thermal Stress. Animals. 2020;10: 430.

doi:10.3390/ani10030430

12. Mullenix GJ, Greene ES, Emami NK, Tellez-Isaias G, Bottje WG, Erf GF, et al. Spirulina platensis Inclusion Reverses Circulating Pro-inflammatory (Chemo)cytokine Profiles in Broilers Fed Low-Protein Diets. Front Vet Sci. 2021;8. doi:10.3389/fvets.2021.640968

13. Farag MR, Alagawany M, Abd El-Hac ME, Dhama K. Nutritional and Healthical Aspects of Spirulina (Arthrospira) for Poultry, Animals and Human. Int J Pharmacol. 2015;12: 36–51. doi:10.3923/ijp.2016.36.51

14. Abdel-Moneim A-ME, Shehata AM, Selim DA, El-Saadony MT, Mesalam NM, Saleh AA. Spirulina platensis and biosynthesized selenium nanoparticles improve performance, antioxidant status, humoral immunity and dietary and ileal microbial populations of heat-stressed broilers. J Therm Biol. 2022;104: 103195. doi:10.1016/j.jtherbio.2022.103195

15. Alghonaim AA, Alqahtani MF, Al-Garadi MA, Suliman GM, Al-Baadani HH, AL-Badwi MA, et al. Effects of different levels of spirulina (Arthrospira platensis) supplementation on productive performance, nutrient digestibility, blood metabolites, and meat quality of growing Najdi lambs. Trop Anim Health Prod. 2022;54: 124. doi:10.1007/s11250-022-03115-9

16. Shi L, Yang R, Yue W, Xun W, Zhang C, Ren Y, et al. Effect of elemental nano-selenium on semen quality, glutathione peroxidase activity, and testis ultrastructure in male Boer goats. Anim Reprod Sci. 2010;118: 248–254. doi:10.1016/j.anireprosci.2009.10.003

17. Hassan F, Mahmoud R, El-Araby I. Growth Performance, Serum Biochemical, Economic Evaluation and IL6 Gene Expression in Growing Rabbits Fed Diets Supplemented with Zinc Nanoparticles. Zagazig Vet J. 2017;45: 238–249. doi:10.21608/zvjz.2017.7949

18. Konkol D, Wojnarowski K. The Use of Nanominerals in Animal Nutrition as a Way to

Improve the Composition and Quality of Animal Products. J Chem. 2018;2018: 1–7. doi:10.1155/2018/5927058

19. Qazi IH, Angel C, Yang H, Zoidis E, Pan B, Wu Z, et al. Role of Selenium and Selenoproteins in Male Reproductive Function: A Review of Past and Present Evidences. Antioxidants. 2019;8: 268. doi:10.3390/antiox8080268

20. Abdelnour SA, Alagawany M, Hashem NM, Farag MR, Alghamdi ES, Hassan FU, et al. Nanominerals: Fabrication Methods, Benefits and Hazards, and Their Applications in Ruminants with Special Reference to Selenium and Zinc Nanoparticles. Animals. 2021;11: 1916. doi:10.3390/ani11071916

21. Shobokshy S, Samaha M, Rheem S, Sahwan F, Wirtu G, Soltan M, et al. Dietary supplementation of nano-selenium eliminates the negative effects of long-term ivermectin injection on growth and reproductive performance of female rabbits. J Adv Vet Anim Res. 2022;9: 128. doi:10.5455/javar.2022.i577

22. Nazıroğlu M, Yıldız K, Tamtürk B, Erturan İ, Flores-Arce M. Selenium and Psoriasis. Biol Trace Elem Res. 2012;150: 3–9. doi:10.1007/s12011-012-9479-5

23. Zhou J, Huang K, Lei XG. Selenium and diabetes—Evidence from animal studies. Free Radic Biol Med. 2013;65: 1548–1556. doi:10.1016/j.freeradbiomed.2013.07.012

24. A. Ali A, T. Hassan S, S. Soliman E. Leverage of Nano-Selenium on Sexual Behavior, Reproductive Performance, Semen Characteristics, and Prophylactics in Rabbit Bucks at Hot Season. Adv Anim Vet Sci. 2021;9. doi:10.17582/journal.aavs/2021/9.11.1908.1918

25. Alagawany M, Qattan SYA, Attia YA, El-Saadony MT, Elnesr SS, Mahmoud MA, et al. Use of Chemical Nano-Selenium as an Antibacterial and Antifungal Agent in Quail Diets and Its Effect on Growth, Carcasses, Antioxidant, Immunity and Caecal Microbes.

Animals. 2021;11: 3027. doi:10.3390/ani11113027

26. Broadway P, Carroll J, Sanchez N. Live Yeast and Yeast Cell Wall Supplements Enhance Immune Function and Performance in Food-Producing Livestock: A Review †,‡. Microorganisms. 2015;3: 417–427. doi:10.3390/microorganisms3030417

27. El-Ratel IT, Gabr AA-W. Effect of Spirulina and Vitamin E on Reproduction and in vitro Embryo Production in Heat-stressed Rabbits. Pakistan J Biol Sci. 2019;22: 545–553. doi:10.3923/pjbs.2019.545.553

28. Moustafa ES, Alsanie WF, Gaber A, Kamel NN, Alaqil AA, Abbas AO. Blue-Green Algae (Spirulina platensis) Alleviates the Negative Impact of Heat Stress on Broiler Production Performance and Redox Status. Animals. 2021;11: 1243. doi:10.3390/ani11051243

29. NRC. Nutrient Requirements of Rabbits,. Washington, D.C.: National Academies Press; 1977. doi:10.17226/35

30. Mostek A, Dietrich MA, Słowińska M, Ciereszko A. Cryopreservation of bull semen is associated with carbonylation of sperm proteins. Theriogenology. 2017;92: 95–102. doi:10.1016/j.theriogenology.2017.01.011

31. Marai IF, Ayyat MS, Abd el-Monem UM. Growth performance and reproductive traits at first parity of New Zealand white female rabbits as affected by heat stress and its alleviation under Egyptian conditions. Trop Anim Health Prod. 2001;33: 451–62. doi:10.1023/a:1012772311177

32. Mendiola JA, Jaime L, Santoyo S, Reglero G, Cifuentes A, Ibañez E, et al. Screening of functional compounds in supercritical fluid extracts from Spirulina platensis. Food Chem. 2007;102: 1357–1367. doi:10.1016/j.foodchem.2006.06.068

33. Alwaleed EA, El-Sheekh M, Abdel-Daim MM, Saber H. Effects of Spirulina platensis and Amphora coffeaeformis as dietary supplements on blood biochemical parameters, intestinal microbial population, and productive performance in broiler chickens. Environ Sci Pollut Res. 2021;28: 1801–1811. doi:10.1007/s11356-020-10597-3

34. El-Ratel I, El-Moghazy M, El-Gaml A, Abu El-Naser I. Effects of Propolis Ethanolic Extract Administration on Quality of Fresh and Cryopreserved Semen, Redox Status, and Sperm Flow Cytometry Parameters of Heat-Stressed Rabbit Bucks. Adv Anim Vet Sci. 2022;10. doi:10.17582/journal.aavs/2022/10.12.2578.2588

35. Chen Z, Zhang JR, Zhou YW, Liang C, Jiang YY. Effect of heat stress on the pituitary and testicular development of Wenchang chicks. Arch Anim Breed. 2015;58: 373–378. doi:10.5194/aab-58-373-2015

36. Peña ST, Gummow B, Parker AJ, Paris DBBP. Antioxidant supplementation mitigates DNA damage in boar (Sus scrofa domesticus) spermatozoa induced by tropical summer. Drevet JR, editor. PLoS One. 2019;14: e0216143. doi:10.1371/journal.pone.0216143

37. Yousef MS, Megahed GA, Abozed GF, Hayder M, Plo HH, Rawy MS. Exogenous gonadotropin-releasing hormone counteracts the adverse effect of scrotal insulation on testicular functions in bucks. Sci Rep. 2022;12: 7869. doi:10.1038/s41598-022-11884-4

38. Parhar IS, Ogawa S, Ubuka T. Reproductive Neuroendocrine Pathways of Social Behavior. Front Endocrinol (Lausanne). 2016;7. doi:10.3389/fendo.2016.00028

39. Walker WH. Molecular mechanisms of testosterone action in spermatogenesis. Steroids. 2009;74: 602–7. doi:10.1016/j.steroids.2008.11.017

40. Abdel-Wareth AAA, Ahmed AE, Hassan HA, Abd El-Sadek MS, Ghazalah AA, Lohakare J. Nutritional impact of nano-selenium, garlic oil, and their combination on growth and

reproductive performance of male Californian rabbits. Anim Feed Sci Technol. 2019;249: 37–45. doi:10.1016/j.anifeedsci.2019.01.016

41. Marković R, Ćirić J, Starčević M, Šefer D, Baltić MŽ. Effects of selenium source and level in diet on glutathione peroxidase activity, tissue selenium distribution, and growth performance in poultry. Anim Heal Res Rev. 2018;19: 166–176. doi:10.1017/S1466252318000105

42. Ewuola E, Akinyemi D. Semen Characteristics of Rabbit Bucks Orally Administered Exogenous L-Selenomethionine. Annu Res Rev Biol. 2017;13: 1–8. doi:10.9734/ARRB/2017/19196

43. Sabés-Alsina M, Tallo-Parra O, Mogas MT, Morrell JM, Lopez-Bejar M. Heat stress has an effect on motility and metabolic activity of rabbit spermatozoa. Anim Reprod Sci. 2016;173: 18–23. doi:10.1016/j.anireprosci.2016.08.004

44. Garner JB, Chamberlain AJ, Vander Jagt C, Nguyen TTT, Mason BA, Marett LC, et al. Gene expression of the heat stress response in bovine peripheral white blood cells and milk somatic cells in vivo. Sci Rep. 2020;10: 19181. doi:10.1038/s41598-020-75438-2

45. Attia YA, El-Hamid AEA, El-Hanoun AM, Al-Harthi MA, Abdel-Rahman GM, Abdella MM. Responses of the Fertility, Semen Quality, Blood Constituents, Immunity and Antioxidant Status of Rabbit Bucks to Type and Magnetizing of Water. Ann Anim Sci. 2015;15: 387–407. doi:10.2478/aoas-2014-0086

46. Surai PF, Kochish II. Nutritional modulation of the antioxidant capacities in poultry: the case of selenium. Poult Sci. 2019;98: 4231–4239. doi:10.3382/ps/pey406

47. Park JH, Lee SI, Kim IH. Effect of dietary Spirulina (Arthrospira) platensis on the growth performance, antioxidant enzyme activity, nutrient digestibility, cecal microflora, excreta

noxious gas emission, and breast meat quality of broiler chickens. Poult Sci. 2018;97: 2451–2459. doi:10.3382/ps/pey093

48. Abdel-Moneim A-ME, Shehata AM, Mohamed NG, Elbaz AM, Ibrahim NS. Synergistic effect of Spirulina platensis and selenium nanoparticles on growth performance, serum metabolites, immune responses, and antioxidant capacity of heat-stressed broiler chickens. Biol Trace Elem Res. 2022;200: 768–779. doi:10.1007/s12011-021-02662-w

49. Khattab AAA, El Basuini MFM, El-Ratel IT, Fouda SF. Dietary probiotics as a strategy for improving growth performance, intestinal efficacy, immunity, and antioxidant capacity of white Pekin ducks fed with different levels of CP. Poult Sci. 2020; 100898. doi:https://doi.org/10.1016/j.psj.2020.11.067

50. Abd El-Hack ME, Alagawany M, Abdelnour S. Responses of growing rabbits to supplementing diet with a mixture of black and red pepper oils as a natural growth promoter. J Anim Physiol Anim Nutr (Berl). 2019;103: 509–517. doi:10.1111/jpn.13045

51. Abdelnour S, Hassan M, Mohammed A, Alhimaidi A, Al-Gabri N, Al-Khaldi K, et al. The Effect of Adding Different Levels of Curcumin and Its Nanoparticles to Extender on Post-Thaw Quality of Cryopreserved Rabbit Sperm. Animals. 2020;10: 1508. doi:10.3390/ani10091508

52. El-Bab AFF, Saghir SAM, El-Naser IAA, El-Kheir SMMA, Abdel-Kader MF, Alruhaimi RS, et al. The Effect of Dietary Saccharomyces cerevisiae on Growth Performance, Oxidative Status, and Immune Response of Sea Bream (Sparus aurata). Life. 2022;12: 1013. doi:10.3390/life12071013

53. Naga Raja Kumari K, Narendra Nath D. Ameliorative measures to counter heat stress in poultry. Worlds Poult Sci J. 2018;74: 117–130. doi:10.1017/S0043933917001003

54. Gershwin ME, Belay A, editors. Spirulina in Human Nutrition and Health. CRC Press; 2007. doi:10.1201/9781420052572

55. Mirzaie S, Zirak-Khattab F, Hosseini SA, Donyaei-Darian H. Effects of dietary Spirulina on antioxidant status, lipid profile, immune response and performance characteristics of broiler chickens reared under high ambient temperature. Asian-Australasian J Anim Sci. 2018;31: 556–563. doi:10.5713/ajas.17.0483

56. Hamza RZ, EL- Megharbel SM, Altalhi T, Gobouri AA, Alrogi AA. Hypolipidemic and hepatoprotective synergistic effects of selenium nanoparticles and vitamin. E against acrylamide- induced hepatic alterations in male albino mice. Appl Organomet Chem. 2020;34. doi:10.1002/aoc.5458

57. Deng R, Chow T-J. Hypolipidemic, antioxidant, and antiinflammatory activities of microalgae Spirulina. Cardiovasc Ther. 2010;28: e33-45. doi:10.1111/j.1755-5922.2010.00200.x

58. Calogero AE, Condorelli RA, Russo GI, La Vignera S. Conservative Nonhormonal Options for the Treatment of Male Infertility: Antibiotics, Anti-Inflammatory Drugs, and Antioxidants. Biomed Res Int. 2017;2017: 1–17. doi:10.1155/2017/4650182

Click here to access/download
Supporting Information
M DATA -FF.xls

Article

The Synergistic Impact of Spirulina and Selenium Nanoparticles Mitigates the Adverse Effects of Heat Stress on Semen Quality of Rabbits BucksThe Synergistic Impact of Spirulina and Selenium Nanoparticles Mitigates the Adverse Effects of Heat Stress on Rabbits Bucks

Ibrahim T. El-Ratel [1], Mawada E. Elbasuny [1], Hamdy A. El-Nagar [2], Abdel-Khalek E. Abdel-Khalek [3], Ali A. El-Raghi [1], Mohammed F. M. El Basuini [4,5,] *, Khaled H. El-Kholy [1], Sara F. Fouda [6]

[1] Department of Animal, Poultry and Fish Production, Faculty of Agriculture, Damietta University, 34517, Damietta, Egypt; Ibrahimtalat@du.edu.eg , mawadaelnabawy@gamil.com , ali21384@yahoo.com , khelkholy@yahoo.com

[2] Department of Biotechnology Research, Animal Production Research Institute, Agricultural Research Center, Giza, Egypt; elnagar1970@hotmail.com

[3] Department of Animal Production, Faculty of Agriculture, Mansoura University, 35516, Mansoura, Egypt; abdelkhalk2004@yahoo.com

[4] Department of Animal Production, Faculty of Agriculture, Tanta University, 31527 Tanta, Egypt; mohamed.elbasuni@agr.tanta.edu.eg

[5] Faculty of Desert Agriculture, King Salman International University, South Sinai, Egypt; mohammed.elbasuini@ksiu.edu.eg

[6] Department of Poultry Production, Faculty of Agriculture, Mansoura University, 35516, Mansoura, Egypt; sarafikry@mans.edu.eg

***** Correspondence: m_fouad_islam@yahoo.com

Abstract: Heat stress has a detrimental effect on animal fertility, particularly testicular functions, including reduced sperm output and quality, which causes an economic loss in the production of rabbits. The present trial investigated the efficacy of dietary Spirulina (SP) (*Arthrospira platensis*),~~-~~ ~~with different levels of~~ selenium nanoparticles (SeNPs), and their combination (SP-SeNPs) on semen quality, haemato-biochemical, oxidative stress, immunity, and sperm ~~fertility~~ quality of heat-stressed (HS) rabbit bucks. Sixty mature bucks (APRI line) were distributed into 6 groups of ten replicates. Bucks in the 1st group were kept under thermoneutral conditions (~~positive~~ control-NC), while the 2nd was kept under heat stress and as a ~~negative~~ control-HS. The control groups were fed a commercial pelleted diet and the other four heat-stressed groups were fed a commercial pelleted diet with 1 g SP, 25 mg SeNPs, 1 g SP+25 mg SeNPs, and 1 g SP+50 mg SeNPs per kg diet, respectively. The dietary inclusion of SP, SeNPs, and their combinations significantly increased hemoglobin, platelets, total serum protein, high-density lipoproteins, glutathione, glutathione peroxidase, superoxide dismutase, and seminal plasma testosterone while decreased triglycerides, total cholesterol, urea, creatinine, and malondialdehyde compared with the ~~negative controls~~control-HS. Red blood cells, packed cell volume, serum albumin, and testosterone significantly increased, while SeNPs, SP+SeNPs25, and SP+SeNPs50 significantly decreased low-density lipoproteins, aspartate, and alanine amino transferees. Total antioxidant capacity substantially increased in serum and seminal plasma, while seminal plasma malondialdehyde decreased in 25 or 50 mg of SeNPs+SP/kg groups. All supplements significantly improved *libido*, sperm livability, concentration, intact acrosome, membrane integrity, total output in fresh semen, and sperm quality in cryopreserved semen. In conclusion, the dietary inclusion of SP plus SeNPs may be a suitable tool for improving reproductive efficiency, health, oxidative stress, and immunity of bucks in the breeding strategy under hot climates.

Keywords: Bucks; Heat Stress; Selenium Nanoparticles; Spirulina; Semen Quality; Sperm Fertility

1. Introduction

Heat stress has a detrimental effect on animal fertility and causes significant economic losses in rabbit production [2–5]. Oxidative stress performs a crucial function in semen quality, quantity, and fertility of sperm cells. Under heat stress (HS), lipid peroxidation increases, particularly when the temperature and humidity exceed the normal ranges [1,2]. HS negatively alters testicular functions and causes DNA fragmentation [3], leading to a reduction in the output and quality of spermatozoa [4] and animal well-being [5]. HS increases sperm abnormality by impairing the mitotic division of spermatocytes during spermatogenesis [1]. These negative impacts of HS on bucks are partially associated with generating free radicals [6]. Spermatozoa of rabbits are rich in metabolic activity and poly-unsaturated fatty acids in their plasma membrane; therefore, a rise in free radicals and lipid peroxidation reduces sperm fertilizing ability [2,7]. Also, HS causes many alterations in blood characteristics, antioxidant capacity, immunity, and reproduction of rabbits [8,9].

In order to lessen the harmful impacts of HS and to avoid buck infertility by limiting ROS production in rabbits, a variety of tools are available. In this way, using natural antioxidants lessens the deleterious impacts of HS on males by assisting with various physiological and metabolic processes [8–11]. Spirulina, a planktonic blue-green alga, exhibits anti-viral, anti-microbial, anti-inflammatory, hepato-protective, and antiparasitic activities and contains antioxidative pigments [12] because it contains a vibrant nutritional profile, particularly proteins, essential amino acids, fatty acids, vitamins, minerals, phytopigments, phycocyanin, polysaccharides, carotenoids, chlorophyll, and phenolic compounds [13–15].

Nano-technology is applied in animal nutritionnuitrition, and the most basic approaches in this area are nanoparticles (NPs) with 1–100 nm dimensions. The NPs are characterized by higher bioavailability, specific surface area, surface center activity, catalytic and adsorption abilities, and less toxic properties [16]. So, elements in NPs form at low levels are more efficient than those in conventional forms when added to diets [17], so NPs can be easily assimilated in the digestive tract [18]. Selenium (Se) is included in several vital physiological processes, like reproductive efficiency, immune status, antioxidant defenses, stress prevention, endocrine, metabolic processes, and is an essential trace element for animals [19–21]. Se is a crucial component in the antioxidant enzyme glutathione peroxidase (GSH-Px) [22,23], which is a significant phase II detoxification enzyme to decrease the level of lipid peroxidation [9]. Dietary Se nanoparticles (SeNPs) supplementation increased the ability of absorption bioavailability, catalytic ability, and surface activity while reducing energy loss [24,25]. In rabbits, SeNPs provide functional benefits like preventive impacts against reproductive toxicity, enhancing the testosterone level, improving the quality of spermatozoa, and decreasing DNA damage of sperm [26].

SeNPs and/or Spirulina (SP) were incorporated to mitigate the negative impacts on the heat-stressed growing rabbit by improving the antioxidant status [14].. It can be used as a promoter of growth, antioxidants, stimulation of immunity, and anti-microbial agents in broilers under HS [14]. SP was reported to be an effective tool under HS conditions for improving buck's reproduction, stimulating antibodies, producing cytokines, increasing antioxidant capacity, and reducing lipid peroxidation [9,27,28]. The impacts of SP, Se, or SeNPs on growth performance are available in rabbits and broilers. However, few studies were carried out on the effect of a combination of SP with different levels of SeNPs on the performance of male rabbits. Thus, this

study aimed to explore the effectiveness of dietary Spirulina (SP) (*Arthrospira platensis*), selenium nanoparticles (SeNPs), and their combination (SP-SeNPs) on semen quality, haemato-biochemical, oxidative stress, immunity, and sperm quality of heat-stressed (HS) rabbit bucks.dietary supplementation of SP with different levels of SeNPs on the semen quality, haemato-biochemical parameters, oxidative stress, immunity, and sperm fertility of rabbit bucks under HS circumstances.

2. Materials and methods

2.1. Animals and management protocol

The study was conducted at a private commercial rabbit farm in Mansoura City, Dakahlia Governorate, Egypt, from December 2021 to April 2022. The experimental animals in this study included 60 sexually mature APRI line bucks (7-8 months of age and 3.15±0.32 kg LBW). Each buck served as a replicate and was kept individually in stainless steel cage batteries (40×50×35cm) accommodated with feeders and automatic drinkers in a closed system with controlled by heat, humidity, and light regimes (12 hrs light: 12 hrs dark) under the same managerial and hygienic conditions. The feeding system of all bucks included a commercial pelleted diet (CPD) based on NRC [29]. The ingredients and chemical composition of CPD are shown in Table 1.

Table 1. Ingredients and chemical composition of the diet (CPD) fed to bucks.

Ingredient	%	Chemical analysis	
Berseem hay	30.05	Total protein %	17.75
Barley	24.60	Crude fiber %	12.38
Wheat bran	21.50	Total lipids %	2.27
Soybean meal (44% CP)	17.50	Digestible Energy Kcal/Kg diet	2500

Molasses	3.00	Ca %	1.24
Dicalcium Phosphate (DCP)	1.60	Na %	0.16
Limestone	0.95	Total phosphorus %	0.80
NaCl	0.30	Lysine %	0.98
Premix *	0.30	Methionine %	0.46
DL-Methionine	0.20	Methionine + Cystine %	0.76

* 1 kg premix contains: Biotin, 0.05 mg; Choline, 250 mg; Folic acid, 3 mg; Niacin, 50 mg; Pantothenic acid, 10 mg; Vitamin B_1, 2 mg; Vitamin B_{12}, 0.01 mg; Vitamin B_6, 2 mg; Vitamin E, 40 mg; Vitamin K_3, 2 mg; Vitamin A, 6000 IU; Vitamin B_2, 4 mg; Vitamin D_3, 900 IU; Co, 0.1 mg; Cu, 5 mg; Fe, 50 mg; I, 0.2 mg; Mn, 85 mg; Se, ~~0,1~~0.1 mg; Zn, 50 mg.

2.2. Design of the experiment

The experimental animals (n=60) were randomly divided into six groups (10/group). Bucks in the 1st group (control-NC, G1) were kept under normal conditions of ambient temperature (11-22 °C) and relative humidity (RH) from 40 to 45% and fed on CPD without supplementation. Bucks in the 2nd group (control-HS, G2) were exposed to HS conditions (32 ± 0.50 °C; 60-66% RH) and fed on CPD without supplementation. Other four treatment groups, including bucks kept under HS conditions as in G2 and fed on CPD supplemented with 1g SP (G3), 25 mg SeNPs (G4), 1 g SP+25 mg SeNPs (G5), and 1 g SP+50 mg SeNPs (G6) per kg diet, respectively. The treatment period was two months, followed by semen collection period of 10 weeks.~~Throughout an experiment of 10 weeks, bucks in the 1st group (positive control, G1) were fed the CPD without supplementation under normal indoor conditions. The average minimum and maximum ambient temperatures were 11 and 22, respectively, and relative humidity (RH) ranged from 40 to 45%. Bucks in the 2nd group (negative control, G2) were fed the CPD without supplementation and daily exposed to HS condition (32 ± 0.50 °C; 60-66% RH) for 6 h (8 a.m.~~

to 2 p.m.) for four days/week, returned to the control condition. Four treatment groups, including bucks in the G3, G4, G5, and G6, were fed the CPD supplemented with 1 g SP, 25 mg SeNPs, 1 g SP+25 mg SeNPs, and 1 g SP+50 mg SeNPs per kg diets, respectively, and were exposed to HS condition.

Treatment of SP was in powder form (Alga Biotechnology Unit, National Research Center, Dokki, Egypt), and SeNPs treatment (Sigma-Aldrich, USA) were involved in this study. The SeNPs morphology by TEM revealed that the mean particle size of SeNPs was 70 nm and the value of ZP was −4.46 mV which showed the best sign of the potential stability of the colloidal system. The mean PDI was 0.13, indicating that nanoparticles produced by this system had the highest homogeneity in size distribution (Figure 1).

2.3. Libido and semen collection

One week before the main semen collection phase (training period), bucks were trained for the semen collection. Sexual desire (libido) was recorded, as a reaction time, as a time in seconds that elapsed from female insertion into the male cage up to the complete ejaculation. Semen was collected by the same veterinarian in the morning (8 a.m.) one/wk. by an artificial vagina (40 - 41°C), and a teaser doe was used. During the experimental period, semen was collected for 10 successive weeks (7 wks. for fresh semen and another 3 wks. for semen cryopreservation).

2.4. Fresh semen evaluation

Semen of 70 ejaculates (10 bucks x 7 weeks) were taken and evaluated in fresh case/group, then an average of each semen parameter/rabbit was calculated. On the day of the semen collection, gel mass (if present) was removed, and net semen volume (NSV) was recorded in the graduated tube connected to the artificial vagina. The pH value was measured with a pH pen. In each

semen sample, the score of mass motility (0-5) was determined; the percentage of forward sperm motility (progressive motility) was examined for about 200 sperm cells in five microscopic fields by using a phase-contrast microscope with a hot stage at 37°C (Leica DM 500, Leica Mikrosysteme Vertrieb GmbH, Wetzlar, Germany).

Percentages of sperm morphological abnormality (head and tail) and livability (live: unstained cells and dead: purple stained cells) were determined in about 200 sperm cells stained by eosin-nigrosine dual stain (5%:10%) using a light microscope at 100 × magnification. Semen was diluted with saline solution (1:99) to determine sperm cell concentration (SCC) with a Neubauer hemocytometer slide (GmbH +Co., Brandstwiete 4, 2000 Hamburg 11, Germany).

The hypo-osmotic swelling test (HOS-t) was employed to measure sperm membrane integrity by incubation (37°C for one hour) of 30 µl semen sample and 300 µl a solution with osmolarity level of 100 mOsm/kg) containing fructose (9 g) and sodium citrate (4.9 g) in 100 ml of distilled water in a water bath [6]. Responses of about 200 spermatozoa to be swollen or with curled tails were determined, and membrane integrity was calculated. Total sperm outputs (TSO) per ejaculate was computed according to the following equations: TSO/ejaculate (10^6/ejaculate) = NSV (ml) x SCC (×106/ml). Acrosomal status, including sperm cells with intact acrosome cap, non-intact acrosome, and sperm cells without acrosome cap, were determined to calculate the percentage of intact acrosome using naphthol yellow S and erythrosine B. [2].

2.5. Semen cryopreservation

In cryopreserved semen (30 ejaculates/group) collected from 10 bucks/group during the last 3 weeks of the experiment, ejaculates without gel mass of each group were pooled, diluted (1 semen: 5 diluents) with Tris-extender. Each 100 ml of the extender contained Tris (3.028 mg),

citric acid (1.675 mg), fructose (1.250 mg), gentamycin (5000 µg), 20% egg yolk, 7% glycerin, and distilled water up to 100 ml. Semen was packed in 0.25 mL straws (IVM technologies, L' Aigle, France) after the dilution process and equilibration (at 5 ◦C for 4 h), then offered at 4-5 cm overhead liquid nitrogen (LN) for 10 min, then preserved in LN at -196 ◦C for 30 days.

Cryopreserved semen straws were thawed at 37◦C for 30 s in a water bath. Post-thawed semen was evaluated for sperm forward motility, livability, abnormality, and membrane integrity as previously conducted in semen at the fresh case.

2.6. Analytical procedures

2.6.1. Blood variables

At the end of the experimental period, 5 bucks in each treatment were randomly selected for blood testing. Blood samples were gathered from the marginal ear-vein of the does after topical anesthetized by Xylocaine 4% anesthetic into heparinized pipes to assess hematological parameters. Other blood samples were collected into non-heparinized tubes and left to clot, then centrifuged at 700 *g* for 20 min at 4°C to separate serum collection, which was stored at −20°C until subsequent analyses of blood biochemicals and antioxidant and immunity markers. Hematological parameters included hemoglobin concentration (Hb), packed-cell volume (PVC), and counts of red blood cells (RBCs), white blood cells (WBCs), and platelets using HB 7021 analyzer. Blood serum biochemical included concentrations of total protein (TP), albumin (AL), triglycerides (TG), total cholesterol (TC), high (HDL) and low-density (LDL) lipoproteins, urea, and creatinine as well as activity of aspartate (AST) and alanine (ALT) amino transferees. Globulin was determined by calculation (the differences between concentration of TP and AL. In

blood serum, levels of total antioxidant capacity (TAC), glutathione (GSH), glutathione peroxidase (GPx), superoxide dismutase (SOD), and malondialdehyde (MDA) were assayed. All biochemicals, enzyme activity, and oxidative capacity studied were assayed colorimetrically by Bio-diagnostic Co. kits (www Bio-diagnostic.com) and UV-VIS Auto spectrophotometer (UV-2602, Labomed, Los Angeles, CA, USA). Also, enzyme-immunoassay of serum testosterone profile was employed using commercial kits (Biosource-Europe S.A. 8, rue de L'Lndustrie. B-1400 Nivelles, Belgium). Coefficients of intra- and inter-assay were 7.8 and 8.4%, respectively. The minimum and maximum detectable limits were 0.1 and 18.0 ng/ml, respectively.

2.6.2. Seminal plasma variables

Samples of fresh semen collected at the last week of the semen collection (the 7th week) from 10 bucks per group were centrifuged (700 *g* for 20 min) to obtain the seminal plasma, which was stored at –20°C) for determining TAC, MDA, and testosterone. Protein carbonyl levels (POC) in sperm media of the post-thawed semen collected during the last semen collection period (the 10th week) were also determined. POC was determined with MBS2600784 protein carbonyl ELISA kit (MyBioSource, Giza, Egypt) based on the manufacturer's technique [30].

2.6.3. Evaluation of sperm ~~Fertility~~ fertility in bucks

Sperm fertility of fresh semen of each group was performed by using 120 sexually mature APRI rabbit females. All does were divided into 6 groups (n = 20), then does in each group naturally mated with 5 bucks/treatment (4 does/buck) during 3 days as a mating interval at the end of the experiment. On days 10-12 post-mating, manual abdominal palpation was performed to determine the pregnancy rate (PR). PR = (positive does (n)/mated does (n) ×100. After parturition, the kindling rate (KR) as numbers of kindled does divided by the number of positive does ×100 was calculated. Also, the litter size of does and viability rate of kits (total born and

alive) were calculated. At weaning (28 days of kit age), litter size and viability rates (live and dead) were recorded.

2.7. Statistical analysis

Statistical analysis comprised the General Linear Model procedures using SAS (Cary, NC, USA: SAS Institute Inc, 2012) to study the effect of treatment by the GLM model: $Y_{ij} = \mu + T_i + e_{ij}$, where Y_{ij}, observations; μ, overall mean; T_i, effect of treatments (i, 1-6); $e_{ij,}$ random error. Tukey's Studentized Range test was used to separate the differences between treatment groups at $P < 0.05$.

3. Results

3.1. Blood hematology and serum biochemistry

Data in Table 2 illustrate the impacts of SP, SeNPs, and their combinations on blood hematology and serum biochemistry. Significant alterations (p < 0.01) in all blood hematology parameters were observed except WBCs (p=0.1538). In comparing control-NC and control-HS ~~positive and negative control~~ groups, the results indicated the harmful effects of HS on all hematological and biochemical variables studied. In comparison with the control-HS group~~negative control group~~, all treatments increased Hb concentration and platelet count (P<0.05). The counts of RBCs and PCV were increased in SeNPs, SP+SeNPs25, and SP+SeNPs50 groups.

All serum biochemical parameters were statistically affected (p<0.001) by the dietary SeNPs enriched with SP (Table 2). All treatments substantially (p<0.05) boosted serum total

protein and HDL, while significantly (P<0.05) decreased TG, TC, urea, and creatinine concentration as compared to the ~~negative~~ control-HS. The concentration of serum AL increased (P<0.0), while LDL, AST, and ALT decreased (P<0.05) in SeNPs, SP+SeNPs25, and SP+SeNPs50 as compared to the ~~negative~~ control-HS and SP groups. The concentration of serum GL significantly (P<0.05) increased in the SP+SeNPs50 group compared to other groups. The control-HS group ~~negative control group~~ exhibited the poorest values of all parameters studied above compared to the treated and control-NC ~~positive control~~ groups.

Table 2. Effect of dietary supplemental Spirulina, selenium nanoparticles, and their combinations on hematological and biochemical parameters of rabbit bucks under heat stress conditions.

Items	Treatments (mg/kg diet)						
	~~Positive~~ Control-NC	~~Negative~~ Control-HS	SP	SeNPs	SP+SeNPs25	SP+SeNPs50	*P*-value
**Hematological variables**							
Hemoglobin (g/dl)	12.5±0.08^{a}	9.18±0.05^{d}	11.93±0.04^{b}	10.5 ±0.14^{c}	12.19 ±0.09^{ab}	12.28± 0.03^{ab}	<0.001
Red blood cells (10^6/mm^3)	6.81±0.09^{a}	5.88± 0.2^{c}	6.10 ±0.06^{bc}	6.26±0.03^{b}	6.35± 0.03^{b}	6.36 ±0.02^{b}	<0.001
White blood cells (10^3/mm^3)	7.38±0.05	7.08 ± 0.05	7.19± 0.02	7.26± 0.03	7.25± 0.02	7.19± 0.02	0.1538
Platelets (10^3/mm^3)	253.1±1.5^{a}	205.02±3.1^{c}	219.24± 3.1^{b}	221.12±3.7^{b}	226.7±1.01^{b}	224.23± 2.2^{b}	<.0001
Packed-cell volume (%)	39.6±1.1^{a}	27.8± 1.5^{d}	30.3±0.93^{cd}	32.9±1.02^{bc}	34.6±0.7^{b}	33.48± 1.4^{bc}	<.0001
**Blood metabolites**							
TP (g/dl)	7.02±0.06^{a}	6.13 ±0.08^{c}	6.28 ±0.03^{b}	6.31± 0.04^{b}	6.40 ± 0.02^{b}	6.41± 0.02^{b}	<0.001
Albumin (g/dl)	4.01±0.034^{a}	3.21±0.015^{c}	3.25±0.02^{bc}	3.26± 0.024^{b}	3.31±0.023^{b}	3.30±0.01^{b}	<0.001
Globulin (g/dl)	3.02±0.09^{ab}	2.92± 0.08^{b}	3.03 ±0.02^{ab}	3.04 ± 0.03^{ab}	3.09 ±0.03^{ab}	3.11± 0.03^{a}	<0.001
Triglycerides (mg/dl)	56.61±1.36^{d}	86.24±1.711^{a}	78.27± 1.15^{b}	79.42 ±0.83^{b}	66.68 ±0.54^{c}	67.95±0.78^{c}	<0.001
Total cholesterol (mg/dl)	83.08±1.6^{c}	114.29 ± 1.8^{a}	97.50±1.7^{b}	96.83± 3.3^{b}	81.98±2.1^{c}	82.95±2.3^{c}	<0.001
High-density lipoproteins (mg/dl)	35.67±1.4^{a}	22.17 ± 1.1^{d}	26.23± 1.3^{c}	28.88± 1.3^{cb}	31.86± 1.3^{ab}	32.13±1.6^{ab}	<0.001
Low-density lipoproteins (mg/dl)	38.95 ±1.1^{b}	50.47 ± 2.3^{a}	47.94 ±2.4^{a}	42.35±1.3^{b}	41.42±1.3^{b}	40.16±1.4^{b}	0.0004
Aspartate amino transferase (IU)	23.65±1.1^{c}	34.32±1.3^{a}	30.23±1.6^{ab}	29.27 ± 1.3^{b}	26.66±1.8^{bc}	27.69±1.4^{bc}	0.0007
Alanine aminotransferase (IU)	20.05±1.8^{c}	28.09±1.3^{a}	26.48 ±0.9^{ab}	23.46 ± 1.4^{bc}	22.73 ±0.95^{cb}	23.15±0.75^{bc}	0.0026
Urea (mg/dl)	28.04±1.5^{c}	39.58 ± 1.9^{a}	34.48±1.2^{b}	34.04 ±1.7^{b}	31.75 ±1.9^{bc}	31.05 ±1.6^{bc}	0.0012
Creatinine (mg/dl)	1.06±0.023^{d}	1.41±0.047^{a}	1.26 ±0.025^{b}	1.18±0.027^{bc}	1.19±0.041^{bc}	1.15±0.042^{cd}	<.0001

Control-NC: control under normal conditions, Control-HS: control under heat stress conditions; SP: spirulina; SeNPs: selenium nanoparticles; SP+SeNPs25: 1g SP + 25 mg SeNPs; SP+SeNPs50: 1g SP+50 mg SeNPs

Values with distinctive superscripts within a row are varied ($P < 0.05$)

3.2. Oxidative status in serum and seminal plasma

The impacts of the dietary Spirulina, selenium nanoparticles, and their interaction on redox status in ~~blood~~ serum are revealed in Table 3. Antioxidant capacity markers were considerably ($P<0.05$) lower in the control-HS ~~negative control~~ group than in the control-NC ~~positive control~~ group, indicating the effects of HS on increasing lipid peroxidation in ~~blood~~ serum and seminal plasma. All dietary supplementations to buck diets increased ($P<0.05$) GSH, GPx, and SOD in serum, while decreasing ($P<0.05$) MDA level as compared to the ~~negative~~ control-HS. Enriching rabbit diets with 25 or 50 mg of SeNPs plus SP/kg diet produced significantly ($P<0.05$) the highest levels of TAC compared to other groups. Concerning the antioxidant activities in the seminal plasma of bucks, TAC increased ($p < 0.05$). At the same time, the MDA level decreased ($P<0.05$) by feeding rabbit diets with 25 or 50 mg of SeNPs plus SP/kg compared to other groups. The ~~negative~~ control-HS group exhibited the lowest activities of all the above antioxidant markers compared to the treated and ~~positive~~ control-NC.

3.3. Libido and semen production

The libido of rabbit bucks was improved ($p<0.05$) by the dietary treatments compared to the ~~negative~~ control-HS group but did not differ from that of the ~~positive~~ control-NC. Mass motility score and progressive motility percentage significantly ($P<0.05$) increased by SeNPs or their combination with SP compared to the ~~negative~~ control-HS and SP groups. All supplements significantly ($P<0.05$) increased sperm livability, SCC, intact acrosome, membrane integrity, and TSO while decreasing abnormality compared with the ~~negative~~ control-HS group. The differences in NSV among the experimental groups were not significant. The most improvement in semen characteristics was by SP+SeNPs50, but still considerably ($P<0.05$) lower than the ~~positive~~ control-NC, except RT and mass motility (Table 4).

Table 3. Effect of dietary supplemental Spirulina, selenium nanoparticles and their combinations on oxidative bio-marks in blood and seminal plasma of rabbit bucks under heat stress conditions.

Items	Treatments (mg/kg diet)						
	~~Positive~~ Control-NC	~~Negative~~ Control-HS	SP	SeNPs	SP+SeNPs25	SP+SeNPs50	*P*-value
**Antioxidant and oxidative markers~~marks~~ in the blood**							
Total antioxidant capacity (nmol/L)	1.94 ± 0.03^{a}	0.86 ± 0.04^{c}	0.95 ± 0.02^{bc}	0.98 ± 0.05^{bc}	1.22 ± 0.08^{b}	1.36 ± 0.08^{b}	<.0001
glutathione (mg/dl)	19.5 ± 0.8^{a}	9.3 ± 0.5^{d}	12 ± 0.4^{c}	13.34 ± 0.4^{c}	15.3 ± 0.8^{b}	15.3 ± 0.5^{b}	<.0001
glutathione peroxidase (IU)	3.53 ± 0.14^{a}	1.42 ± 0.08^{d}	2.06 ± 0.08^{c}	2.28 ± 0.09^{bc}	2.37 ± 0.03^{b}	2.44 ± 0.04^{b}	<.0001
superoxide dismutase (IU)	32.3 ± 1.04^{a}	19.4 ± 0.8^{c}	22.8 ± 0.6^{b}	23.6 ± 1.6^{b}	23.8 ± 0.5^{b}	24.5 ± 0.8^{b}	<.0001
malondialdehyde (nmol/mL)	4.29 ± 0.3^{c}	8.13 ± 0.5^{a}	6.24 ± 0.1^{b}	6.22 ± 0.1^{b}	6.17 ± 0.1^{b}	6.14 ± 0.05^{b}	<.0001
**Antioxidant and oxidative markers~~marks~~ in the seminal plasma**							
Total antioxidant capacity (nmol/L)	22.65 ± 1.3^{a}	10.01 ± 1.8^{c}	10.62 ± 1.02^{c}	12.85 ± 0.68^{bc}	15.1 ± 0.82^{b}	15.24 ± 0.71^{b}	<.0001
malondialdehyde (nmol/mL)	35.06 ± 2.9^{b}	51.88 ± 1.9^{a}	50.56 ± 1.7^{a}	46.4 ± 1.2^{ab}	45.91 ± 1.5^{b}	45.8 ± 1.05^{b}	0.0002

Control-NC: control under normal conditions, Control-HS: control under heat stress conditions; SP: spirulina; SeNPs: selenium nanoparticles; SP+SeNPs25: 1g SP + 25 mg SeNPs; SP+SeNPs50: 1g SP+50 mg SeNPs. Values with distinctive superscripts within a row are varied ($P < 0.05$)

Table 4. Effect of dietary supplemental Spirulina, selenium nanoparticles and their combinations on semen ~~production~~ quality parameters of rabbit bucks under heat stress conditions.

Items	~~Positive~~ Control-NC	Treatments (mg/kg diet)					
		~~Negative~~ Control-HS	SP	SeNPs	SP+SeNPs25	SP+SeNPs50	*P*-value
Reaction time (sec.)	14.0 ±0.7^{c}	23.7 ± 0.6^{a}	20.8±1.3^{b}	19.2±1.1^{b}	14.3±0.7^{c}	13.9±0.6^{c}	<.0001
Net semen volume (ml)	0.75 ±0.05	0.55±0.02	0.66±0.02	0.64±0.03	0.65±0.03	0.67±0.02	0.069
Mass motility	3.8±0.2^{a}	2.6±0.16^{c}	2.9±0.2^{bc}	3.3±0.15^{ab}	3.5±0.17^{a}	3.5±0.17^{a}	0.0002
Progressive motility (%)	82.5±2.0^{a}	55 ± 2.6^{d}	75±3.4^{cd}	63.5±2.6^{bc}	66.5±3.3^{b}	70.5± 2.3^{b}	<.0001
Sperm livability (%)	85.9±1.8^{a}	51.1±1.6^{e}	63.4±1.03^{d}	68.5± 1.7^{c}	71.7±1.17^{bc}	72.8±0.8^{b}	<.0001
Sperm abnormality (%)	11.8 ±0.9^{c}	24.9±0.9^{a}	21.2±1.05^{b}	20.8±1.05^{b}	20.4± 1.7^{b}	18.2±0.9^{b}	<.0001
Sperm cell concentration (10^6/ml)	356.1±2.2^{a}	196.5±5.7^{f}	224.8± 3.2^{e}	254.3±3.5^{d}	285.5±3.9^{c}	296.8±2.6^{b}	<.0001
Intact acrosome (%)	89.4 ±1.6^{a}	60.9±1.04^{e}	74.8± 1.2^{d}	73.7±0.9^{d}	78.2± 0.8^{c}	83.8±0.9^{b}	<.0001
Membrane integrity (%)	45.1 ±1.4^{a}	21.3 ±0.7^{d}	26.1±0.9^{c}	26.7± 0.6^{c}	36.7± 1.04^{b}	37.1±0.8^{b}	<.0001
Total Sperm output (10^6/ejaculate)	266.6±17.9^{a}	107.4±4.9^{e}	148.7±4.3^{d}	162.8± 6.1^{cd}	185.1±9.1^{bc}	200.09±6.7^{b}	<.0001

Control-NC: control under normal conditions, Control-HS: control under heat stress conditions; SP: spirulina; SeNPs: selenium nanoparticles; SP+SeNPs25: 1g SP + 25 mg SeNPs; SP+SeNPs50: 1g SP+50 mg SeNPs. Values with distinctive superscripts within a row are varied ($P < 0.05$)

3.4. Sperm Quality in Post-Thawed Semen

The effects of dietary treatment supplements on sperm properties in post-thawed semen are presented in Table 5. The present results indicated that the dietary SP, SeNPs, and their combination supplements significantly (*P* <0.05) enhanced sperm quality in cryopreserved semen in terms of increasing SPM, SL, and MI and decreased sperm abnormality and PCO in comparison with the ~~negative~~ control-HS. It is of interest to note the maximum improvement occurred in sperm characteristics after thawing by SP+SeNPs50 as compared to the ~~negative~~ control-HS still to be lower than that exhibited in the ~~positive~~ control-NC group.

3.5. Testosterone in ~~blood~~ serum and seminal plasma

Results illustrated in Table 6 showed that testosterone level was significantly (*P* <0.05) increased by SeNPs and their combinations in blood serum with SP, while significantly (*P* <0.05) increased in the seminal plasma by all supplements.

3.6. Sperm fertility

The dietary treatment of bucks used in natural mating of does with both SP plus SeNPs significantly ($p<0.05$) increased pregnancy rate (PR) and delivered rate (DR) as compared to the ~~negative~~ control-HS other treatments, but PR and DR were similar to that in the ~~positive~~ control-NC group. SeNPs and their combinations with SP significantly ($P<0.05$) improved litter size at birth (Total and live) and weaning, and viability rate only at birth (Table 7).

Table 5. Effect of dietary supplemental Spirulina, selenium nanoparticles and their combinations on sperm variables in post-thawed heat-stressed rabbit semen.

Items	~~Positive~~ Control-NC	Treatments (mg/kg diet)					
		~~Negative~~ Control-HS	SP	SeNPs	SP+SeNPs25	SP+SeNPs50	*P*-value
Progressive motility (%)	72.4± 1.2^{a}	35.1±0.8^{e}	41.2±1.1^{d}	55.5±1.5^{c}	56.4±1.5^{c}	60.9±1.4^{b}	<.0001
Sperm livability (%)	68.9±1.4^{a}	42.3±0.9^{d}	54.6±0.6^{c}	54.6±1.4^{c}	61.5±0.8^{b}	62.8±0.8^{b}	<.0001
Sperm abnormality (%)	13.1±0.7^{d}	26.9± 0.8^{a}	24.5± 0.7^{b}	22.1±0.9^{b}	21.8±1.1^{cb}	21.5±1.2^{c}	<.0001
Membrane integrity (%)	73.5±0.8^{a}	40.7±0.9^{e}	47.4±1.03^{d}	56.8±1.2^{c}	58.2±1.1^{c}	62.30±1.09^{b}	<.0001
protein carbonyl (ng/mg)	2.38± 0.04^{c}	4.04±0.06^{a}	3.80± 0.05^{b}	3.66± 0.12^{b}	3.63± 0.06^{b}	3.58 ± 0.08^{b}	<.0001

Control-NC: control under normal conditions, Control-HS: control under heat stress conditions; SP: spirulina; SeNPs: selenium nanoparticles; SP+SeNPs25: 1g SP + 25 mg SeNPs; SP+SeNPs50: 1g SP+50 mg SeNPs. Values with distinctive superscripts within a row are varied ($P < 0.05$)

Table 6. Effect of dietary supplemental Spirulina, selenium nanoparticles and their combinations on blood and seminal testosterone of rabbit bucks under heat stress conditions.

Testosterone (ng/ml)	~~Positive~~ Control-NC	Treatments (mg/kg diet)					
		~~Negative~~ Control-HS	SP	SeNPs	SP+SeNPs25	SP+SeNPs50	*P*-value
Serum	3.65±0.053^{a}	2.09 ± 0.029^{c}	2.15±0.052^{c}	2.26±0.019^{b}	2.26±0.016^{b}	2.29±0.017 ^{b}	<.0001
Seminal Plasma	3.18±0.033^{a}	2.02±0.067^{d}	2.05±0.057^{cd}	2.17±0.024^{bc}	2.21± 0.019 ^{b}	2.26±0.024^{b}	<.0001

Control-NC: control under normal conditions, Control-HS: control under heat stress conditions; SP: spirulina; SeNPs: selenium nanoparticles; SP+SeNPs25: 1g SP + 25 mg SeNPs; SP+SeNPs50: 1g SP+50 mg SeNPs. Values with distinctive superscripts within a row are varied ($P < 0.05$).

Table 7. Reproductive performance of rabbits does mate by rabbit bucks treated Spirulina, selenium nanoparticles, and their combinations under heat stress conditions.

**Items**	Treatments (mg/kg diet)						
	**~~Positive~~ Control-NC**	**~~Negative~~ Control-HS**	**SP**	**SeNPs**	**SP+SeNPs25**	**SP+SeNPs50**	***P*-value**
Total mated rabbit does (n)	30	30	30	30	30	30	--
Pregnant rabbit does (n, %)	27/30 (90)^{a}	17 (56.67)^{b}	19 (63.33)^{b}	19 (63.33)^{b}	25 (83.33)^{a}	26 (86.67)^{a}	--
Delivered rabbit does (n, %)	27/27 (100)^{a}	13 (76.47)^{b}	15 (78.94)^{b}	16 (84.21)^{ab}	23 (92.00)^{a}	25 (96.15)^{a}	--
Total litter size at birth (n)	9.04 ±0.37^{a}	5.54±0.38^{e}	5.87± 0.27^{de}	6.81±0.41^{cd}	7.65±0.27^{bc}	8.04±0.23^{b}	<.0001
Live litter size at birth (n)	8.7±0.3^{a}	4.3±0.31^{d}	5.07±0.35^{d}	6.0± 0.4^{c}	6.96± 0.24^{b}	7.44± 0.24^{b}	<.0001
Viability rate at birth (%)	96.7± 1.1^{a}	78.8± 4.6^{c}	85.44±3.5^{bc}	88.5± 2.6^{b}	91.4± 1.8^{ab}	92.68±1.9^{ab}	--
Litter size at weaning (n)	8.33 ±0.33^{a}	3.92± 0.33^{d}	4.67± 0.29^{d}	5.63± 0.33^{c}	6.65± 0.25^{b}	7.12± 0.21^{b}	<.0001
Viability rate at weaning (%)	95.80± 1.3	91.79±3.05	93.75± 2.86	94.916± 2.16	95.70 ± 1.42	96.21± 1.30	--

Control-NC: control under normal conditions, Control-HS: control under heat stress conditions; SP: spirulina; SeNPs: selenium nanoparticles; SP+SeNPs25: 1g SP + 25 mg SeNPs; SP+SeNPs50: 1g SP+50 mg SeNPs. Values with distinctive superscripts within a row are varied ($P < 0.05$).

4. Discussion

Heat stress has an undesirable results on the health and fertility of animals and leads to substantial economic losses in the production of rabbits [2-5]. In subtropical regions, the harmful effects of HS on livestock are not easy to mitigate, especially when the high ambient temperature is combined with high humidity. In the present study, the temperature–humidity index (THI) indicated that rabbits in treatment and ~~negative~~ control-HS groups are exposed to severe heat stress (28.9 to <30.0) [31]. The positive effects of SP or Se in diets of rabbits or broilers under HS conditions, and dietary SP supplementation, as a functional component, due to its high bio-active components and its functional properties have previously been reported [32,33]. To our knowledge, the present study is the 1st to illustrate the cooperation of SP with different levels of SeNPs on the semen characteristics of heat-stressed bucks.

In the current study, the dramatic reduction observed in the quality and function of buck spermatozoa by HS was reported by El-Ratel et al. [34]. HS condition inhibits hypothalamic gonadotropin-releasing hormone (GnRH) synthesis and secretion. The function of the testis, testosterone production, and the quality of rabbit semen were mainly affected by GnRH [35]. As proved in our study, HS negatively influences the motility and membrane integrity of bucks in the control-HS compared with the control-NC group due to the ROS generation in the testicular tissues of bucks, leading to lipid peroxidation (antioxidant defense system damage) in the sperm plasma membrane [36]. The obtained results indicated the deleterious impact of HS on reducing testosterone secretion in bucks of ~~negative~~ control-HS group via a reduction in testosterone production as a result of destroying the function of Leydig cells [37]. The RT is controlled by the hypothalamus–pituitary–testis axis, nervous system, hormones, and sexual pheromones [38]. Therefore, enhancing testosterone biosynthesis [3,27] improved the sexual desire of bucks in all

treatment groups by decreasing RT compared with the ~~negative~~ control-HS group. Testosterone is required for spermatogenesis and maturation of sperm cells [39], leading to a marked reduction in abnormality and acrosomal damage. In association with enhancing the testosterone profile, antioxidant administration (SP, SeNPs, or their combinations) improved, quantitatively and qualitatively, the semen production, including mass motility score, and the percentages of sperm progressive motility, livability, normality, acrosome intact and integrity of membrane, SCC, and TSO which adversely affected by HS in rabbits [2,3]. The pronounced improvement in semen characteristics in our study was by SP+SeNPs50, but still lower ($P<0.05$) than those of bucks in the ~~positive~~ control-NC, except RT and mass motility score. In this context, a water supplementation of SeNPs significantly improved semen characteristics in rabbit bucks [24]. Se supplementations had beneficial actions via increasing serum testosterone concentrations, enhanced semen quality, and reduced sperm DNA damage in rabbit bucks [40]. These improvements may be due to the structure and bioavailability of SeNPs molecules versus the inorganic form of Se to cover the needs of the testis and epididymal segments from Se required for spermatogenesis, consequently, high-fertile sperm production and high-quality semen [24]. Supplementation of organic Se also enhanced the quality of semen and seminal plasma of heat-stressed rabbit bucks by improving the accessory sex gland functions [3]. Also, the motility, livability, and SCC of sperm were enhanced by Se treatments [41,42].

As a result, we addressed the positive impacts on fresh semen quality of bucks treated with SeNPs enriched with SP combinations, but the main question 'Are bucks with these treatments, particularly during hot climates, have profits on sperm freezing and fertilizing capabilities after cryopreservation? The outcomes in this context showed that rabbit bucks with different levels of SeNP+SP combinations substantially improved sperm characteristics in post-

thawed semen. This improvement concerns good quality semen produced by bucks in treatment groups. In rabbits, sperm characteristics in cryopreserved semen are associated with their characteristics before cryopreservation.

Antioxidant enzymes are reduced when bucks are exposed to HS [43]. Increasing MDA level causes organ damage in rabbits [44] in association with lower reproductive performance of bucks [2]. The harmful effects of HS on antioxidant enzymes and MDA levels were observed for bucks in the ~~negative~~ control-HS in comparison with the ~~positive~~ control-NC group. Therefore, decreasing oxidative stress is a crucial means of mitigating heat-stressed rabbits. The enhancement in the semen quality of bucks in treated groups in the current study was mainly related to the properties of either SP [15] or Se [3] alone and/or their synergetic effects, as antioxidants, which protect the cells from damage by enhancing antioxidant enzyme (GSH, GPx, and SOD) activity as well as reducing MDA level in serum and seminal plasma. The antioxidants scavenge ROS in the testes to decrease the endogenous oxidative damage by increasing antioxidant enzyme activities [45]. The antioxidant enzyme, SOD, converts superoxide anions to H_2O_2, which GPx catalyzes to H_2O or alcohol [46]. The reduction in MDA production by SP and SeNPs combinations mitigated the oxidative stress condition. SP contains β-carotene, Se, polypeptides, pigments, tocopherol, and phenols. Phycocyanin, the primary pigment in SP, plays a vital role in potent antioxidant effects [47,48]. In this context, both enzymatic and non-enzymatic antioxidant defense mechanisms were affected positively by Se addition in the diet of poultry [46].

It is worth noting that SeNPs enriched with SP at levels of 0.25 and 50 mg/kg diet improved the hematological blood parameters. A hematological and biochemical blood parameter represents the animal's health [49]. Considering the findings of our study, SeNPs

enriched with SP showed a significant increase in Hb, RBCs, PLT, and PCV compared to the ~~negative~~ control-HS group because the dietary photogenic, as an immune-modulatory agent, produced a significantly higher erythrogram than a control group in heat-stressed rabbits [20,50,51]. This improvement may indicate that treating SeNPs enriched with SP can protect rabbits from anemia under HS conditions [52].

Our results indicated deleterious effects of HS on all hematological parameters studied in terms of decreasing Hb concentration, RBCs and platelets count, and PCV ($P<0.05$) in control-Hs as compared to control-NC. It is worth noting that SeNPs enriched with SP at levels of 0.25 and 50 mg/kg diet improved the hematological blood parameters. A hematological and biochemical blood parameter represents the animal's health [49]. Considering the findings of our study, SeNPs enriched with SP showed a significant increase in Hb, RBCs, PLT, and PCV compared to the control-HS group because the dietary photogenic, as an immune-modulatory agent, produced a significantly higher erythrogram than a control group in heat-stressed rabbits [20,50,51]. This improvement may indicate that treating SeNPs enriched with SP can protect rabbits from anemia under HS conditions [52].

In our study, HS reduced total protein and increased TC concentrations in the serum of bucks in the ~~negative~~ control~~s~~-HS, which agrees with the results of Liang et al. [9], who found a decrease in total protein and an increase in TC in heat-stressed rabbits. HS condition elevates the secretion of glucocorticoid, which stimulates gluconeogenesis [53]. The observed improvement of serum total protein and its main components (albumin and globulin) in association with reducing urea and creatinine levels in the serum of bucks in treatment groups compared to the ~~negative~~ control-HS may indicate improving protein utilization of treated bucks under HS. Also, the contents of proteins, essential amino acids, minerals, vitamins, phospholipids, and

antioxidants in SP may cause improvement in protein utilization [13,54]. The hypolipidemic activities of SP, SeNPs, or their combinations in reducing TC and TG, and increasing HDL concentration in serum were reported in animals and chickens [55,56]. Such effect may be attributed to the fact that all supplements could decrease absorbed and synthesized cholesterol in the poultry gut [50]. In the same context, the polyphenolic contents in SP could inhibit pancreatic lipase activity and decrease the lipids in blood [57].

The results showed that AST and ALT activities increased in bucks exposed to HS in the ~~negative ~~control-HS~~s~~. Similarly, Maria et al. [43] reported that HS showed damage and inflammation in the liver. According to the present results in our study, reducing AST and ALT activity as well as urea and creatinine in the serum of bucks in treatment groups may suggest a safe use of SP, SeNPs, or their combination on the normal liver and kidney functions of bucks.

In rabbits, semen variables are the significant factors affecting the reproductive performance of doe rabbits [7]. Increasing the percentages of motility and normality, as well as concentrations of spermatozoa, is associated with an enhancement in fertility [2]. Additionally, sperm motility percentage affected the number of kits of doe rabbits. In mammals, sperm characteristics are essential in determining sperm fertility. The present study showed higher sperm fertility in a bucks-fed diet supplemented with SP+SeNPs combinations due to the protection of spermatozoa from lipid peroxidation, which decrease the injury and damage of sperm cells, resulting in an enhancement in male fertility [3,45,58].

Conclusion

Dietary inclusion of *spirulina platensis*, selenium nanoparticles, or their combinations (SP-SeNPs) could be potentially used as potent antioxidants for improving the reproductive

performance of rabbit bucks by eliminating the deleterious effects of the oxidative stress under heat stress conditions via enhancing sexual desire, semen characteristics, fertilizing ability, health and antioxidant statuses, and immunity of rabbit bucks. The combinations of Spirulina (1 g) plus each level of SeNPs (25 or 50 mg) supplemented to the diet provides the most benefits, which can be recommended as a valuable tool for farming rabbit bucks utilized in natural mating or artificial insemination under heat stress circumstances.

References

1. Attia YA, El-Naggar AS, Abou-Shehema BM, Abdella AA. Effect of Supplementation with Trimethylglycine (Betaine) and/or Vitamins on Semen Quality, Fertility, Antioxidant Status, DNA Repair and Welfare of Roosters Exposed to Chronic Heat Stress. Animals. 2019;9: 547. doi:10.3390/ani9080547
2. El-Ratel IT, Attia KAH, El-Raghi AA, Fouda SF. Relief of the negative effects of heat stress on semen quality, reproductive efficiency and oxidative capacity of rabbit bucks using different natural antioxidants. Anim Biosci. 2021;34: 844–854. doi:10.5713/ajas.20.0258
3. Hosny NS, Hashem NM, Morsy AS, Abo-elezz ZR. Effects of Organic Selenium on the Physiological Response, Blood Metabolites, Redox Status, Semen Quality, and Fertility of Rabbit Bucks Kept Under Natural Heat Stress Conditions. Front Vet Sci. 2020;7. doi:10.3389/fvets.2020.00290
4. Türk G, Çeribaşı AO, Şimşek ÜG, Çeribaşı S, Güvenç M, Özer Kaya Ş, et al. Dietary rosemary oil alleviates heat stress-induced structural and functional damage through lipid peroxidation in the testes of growing Japanese quail. Anim Reprod Sci. 2016;164: 133–

143. doi:10.1016/j.anireprosci.2015.11.021

5. Ayo JO, Obidi JA, Rekwot PI. Effects of Heat Stress on the Well-Being, Fertility, and Hatchability of Chickens in the Northern Guinea Savannah Zone of Nigeria: A Review. ISRN Vet Sci. 2011;2011: 1–10. doi:10.5402/2011/838606

6. El-Desoky NI, Hashem NM, Elkomy A, Abo-elezz ZR. Physiological response and semen quality of rabbit bucks supplemented with Moringa leaves ethanolic extract during summer season. Animal. 2017;11: 1549–1557. doi:10.1017/S1751731117000088

7. Attia YA, Hamed RS, Bovera F, Abd El-Hamid AE-HE, Al-Harthi MA, Shahba HA. Semen quality, antioxidant status and reproductive performance of rabbits bucks fed milk thistle seeds and rosemary leaves. Anim Reprod Sci. 2017;184: 178–186. doi:10.1016/j.anireprosci.2017.07.014

8. Kholy K, Wafa W, Nagar H, Aboelmagd A, Ratel I. Physiological response, testicular function, and health indices of rabbit males fed diets containing phytochemicals extract under heat stress conditions. J Adv Vet Anim Res. 2021;8: 256. doi:10.5455/javar.2021.h510

9. Liang Z-L, Chen F, Park S, Balasubramanian B, Liu W-C. Impacts of Heat Stress on Rabbit Immune Function, Endocrine, Blood Biochemical Changes, Antioxidant Capacity and Production Performance, and the Potential Mitigation Strategies of Nutritional Intervention. Front Vet Sci. 2022;9: 906084. doi:10.3389/fvets.2022.906084

10. Abdel-Hady SB. Effect of dietary protein levels on the productive and reproductive performance of rabbits., 11: 167-177. Egypt J Rabbit Sci. 2001;11: 167–177.

11. Sheiha AM, Abdelnour SA, Abd El-Hack ME, Khafaga AF, Metwally KA, Ajarem JS, et al. Effects of Dietary Biological or Chemical-Synthesized Nano-Selenium

Supplementation on Growing Rabbits Exposed to Thermal Stress. Animals. 2020;10: 430. doi:10.3390/ani10030430

12. Mullenix GJ, Greene ES, Emami NK, Tellez-Isaias G, Bottje WG, Erf GF, et al. Spirulina platensis Inclusion Reverses Circulating Pro-inflammatory (Chemo)cytokine Profiles in Broilers Fed Low-Protein Diets. Front Vet Sci. 2021;8. doi:10.3389/fvets.2021.640968

13. Farag MR, Alagawany M, Abd El-Hac ME, Dhama K. Nutritional and Healthical Aspects of Spirulina (Arthrospira) for Poultry, Animals and Human. Int J Pharmacol. 2015;12: 36–51. doi:10.3923/ijp.2016.36.51

14. Abdel-Moneim A-ME, Shehata AM, Selim DA, El-Saadony MT, Mesalam NM, Saleh AA. Spirulina platensis and biosynthesized selenium nanoparticles improve performance, antioxidant status, humoral immunity and dietary and ileal microbial populations of heat-stressed broilers. J Therm Biol. 2022;104: 103195. doi:10.1016/j.jtherbio.2022.103195

15. Alghonaim AA, Alqahtani MF, Al-Garadi MA, Suliman GM, Al-Baadani HH, AL-Badwi MA, et al. Effects of different levels of spirulina (Arthrospira platensis) supplementation on productive performance, nutrient digestibility, blood metabolites, and meat quality of growing Najdi lambs. Trop Anim Health Prod. 2022;54: 124. doi:10.1007/s11250-022-03115-9

16. Shi L, Yang R, Yue W, Xun W, Zhang C, Ren Y, et al. Effect of elemental nano-selenium on semen quality, glutathione peroxidase activity, and testis ultrastructure in male Boer goats. Anim Reprod Sci. 2010;118: 248–254. doi:10.1016/j.anireprosci.2009.10.003

17. Hassan F, Mahmoud R, El-Araby I. Growth Performance, Serum Biochemical, Economic Evaluation and IL6 Gene Expression in Growing Rabbits Fed Diets Supplemented with Zinc Nanoparticles. Zagazig Vet J. 2017;45: 238–249. doi:10.21608/zvjz.2017.7949

18. Konkol D, Wojnarowski K. The Use of Nanominerals in Animal Nutrition as a Way to Improve the Composition and Quality of Animal Products. J Chem. 2018;2018: 1–7. doi:10.1155/2018/5927058

19. Qazi IH, Angel C, Yang H, Zoidis E, Pan B, Wu Z, et al. Role of Selenium and Selenoproteins in Male Reproductive Function: A Review of Past and Present Evidences. Antioxidants. 2019;8: 268. doi:10.3390/antiox8080268

20. Abdelnour SA, Alagawany M, Hashem NM, Farag MR, Alghamdi ES, Hassan FU, et al. Nanominerals: Fabrication Methods, Benefits and Hazards, and Their Applications in Ruminants with Special Reference to Selenium and Zinc Nanoparticles. Animals. 2021;11: 1916. doi:10.3390/ani11071916

21. Shobokshy S, Samaha M, Rheem S, Sahwan F, Wirtu G, Soltan M, et al. Dietary supplementation of nano-selenium eliminates the negative effects of long-term ivermectin injection on growth and reproductive performance of female rabbits. J Adv Vet Anim Res. 2022;9: 128. doi:10.5455/javar.2022.i577

22. Nazıroğlu M, Yıldız K, Tamtürk B, Erturan İ, Flores-Arce M. Selenium and Psoriasis. Biol Trace Elem Res. 2012;150: 3–9. doi:10.1007/s12011-012-9479-5

23. Zhou J, Huang K, Lei XG. Selenium and diabetes—Evidence from animal studies. Free Radic Biol Med. 2013;65: 1548–1556. doi:10.1016/j.freeradbiomed.2013.07.012

24. A. Ali A, T. Hassan S, S. Soliman E. Leverage of Nano-Selenium on Sexual Behavior, Reproductive Performance, Semen Characteristics, and Prophylactics in Rabbit Bucks at Hot Season. Adv Anim Vet Sci. 2021;9. doi:10.17582/journal.aavs/2021/9.11.1908.1918

25. Alagawany M, Qattan SYA, Attia YA, El-Saadony MT, Elnesr SS, Mahmoud MA, et al. Use of Chemical Nano-Selenium as an Antibacterial and Antifungal Agent in Quail Diets

and Its Effect on Growth, Carcasses, Antioxidant, Immunity and Caecal Microbes. Animals. 2021;11: 3027. doi:10.3390/ani11113027

26. Broadway P, Carroll J, Sanchez N. Live Yeast and Yeast Cell Wall Supplements Enhance Immune Function and Performance in Food-Producing Livestock: A Review †,‡. Microorganisms. 2015;3: 417–427. doi:10.3390/microorganisms3030417

27. El-Ratel IT, Gabr AA-W. Effect of Spirulina and Vitamin E on Reproduction and in vitro Embryo Production in Heat-stressed Rabbits. Pakistan J Biol Sci. 2019;22: 545–553. doi:10.3923/pjbs.2019.545.553

28. Moustafa ES, Alsanie WF, Gaber A, Kamel NN, Alaqil AA, Abbas AO. Blue-Green Algae (Spirulina platensis) Alleviates the Negative Impact of Heat Stress on Broiler Production Performance and Redox Status. Animals. 2021;11: 1243. doi:10.3390/ani11051243

29. NRC. Nutrient Requirements of Rabbits,. Washington, D.C.: National Academies Press; 1977. doi:10.17226/35

30. Mostek A, Dietrich MA, Słowińska M, Ciereszko A. Cryopreservation of bull semen is associated with carbonylation of sperm proteins. Theriogenology. 2017;92: 95–102. doi:10.1016/j.theriogenology.2017.01.011

31. Marai IF, Ayyat MS, Abd el-Monem UM. Growth performance and reproductive traits at first parity of New Zealand white female rabbits as affected by heat stress and its alleviation under Egyptian conditions. Trop Anim Health Prod. 2001;33: 451–62. doi:10.1023/a:1012772311177

32. Mendiola JA, Jaime L, Santoyo S, Reglero G, Cifuentes A, Ibañez E, et al. Screening of functional compounds in supercritical fluid extracts from Spirulina platensis. Food Chem.

2007;102: 1357–1367. doi:10.1016/j.foodchem.2006.06.068

33. Alwaleed EA, El-Sheekh M, Abdel-Daim MM, Saber H. Effects of Spirulina platensis and Amphora coffeaeformis as dietary supplements on blood biochemical parameters, intestinal microbial population, and productive performance in broiler chickens. Environ Sci Pollut Res. 2021;28: 1801–1811. doi:10.1007/s11356-020-10597-3

34. El-Ratel I, El-Moghazy M, El-Gaml A, Abu El-Naser I. Effects of Propolis Ethanolic Extract Administration on Quality of Fresh and Cryopreserved Semen, Redox Status, and Sperm Flow Cytometry Parameters of Heat-Stressed Rabbit Bucks. Adv Anim Vet Sci. 2022;10. doi:10.17582/journal.aavs/2022/10.12.2578.2588

35. Chen Z, Zhang JR, Zhou YW, Liang C, Jiang YY. Effect of heat stress on the pituitary and testicular development of Wenchang chicks. Arch Anim Breed. 2015;58: 373–378. doi:10.5194/aab-58-373-2015

36. Peña ST, Gummow B, Parker AJ, Paris DBBP. Antioxidant supplementation mitigates DNA damage in boar (Sus scrofa domesticus) spermatozoa induced by tropical summer. Drevet JR, editor. PLoS One. 2019;14: e0216143. doi:10.1371/journal.pone.0216143

37. Yousef MS, Megahed GA, Abozed GF, Hayder M, Plo HH, Rawy MS. Exogenous gonadotropin-releasing hormone counteracts the adverse effect of scrotal insulation on testicular functions in bucks. Sci Rep. 2022;12: 7869. doi:10.1038/s41598-022-11884-4

38. Parhar IS, Ogawa S, Ubuka T. Reproductive Neuroendocrine Pathways of Social Behavior. Front Endocrinol (Lausanne). 2016;7. doi:10.3389/fendo.2016.00028

39. Walker WH. Molecular mechanisms of testosterone action in spermatogenesis. Steroids. 2009;74: 602–7. doi:10.1016/j.steroids.2008.11.017

40. Abdel-Wareth AAA, Ahmed AE, Hassan HA, Abd El-Sadek MS, Ghazalah AA, Lohakare

J. Nutritional impact of nano-selenium, garlic oil, and their combination on growth and reproductive performance of male Californian rabbits. Anim Feed Sci Technol. 2019;249: 37–45. doi:10.1016/j.anifeedsci.2019.01.016

41. Marković R, Ćirić J, Starčević M, Šefer D, Baltić MŽ. Effects of selenium source and level in diet on glutathione peroxidase activity, tissue selenium distribution, and growth performance in poultry. Anim Heal Res Rev. 2018;19: 166–176. doi:10.1017/S1466252318000105

42. Ewuola E, Akinyemi D. Semen Characteristics of Rabbit Bucks Orally Administered Exogenous L-Selenomethionine. Annu Res Rev Biol. 2017;13: 1–8. doi:10.9734/ARRB/2017/19196

43. Sabés-Alsina M, Tallo-Parra O, Mogas MT, Morrell JM, Lopez-Bejar M. Heat stress has an effect on motility and metabolic activity of rabbit spermatozoa. Anim Reprod Sci. 2016;173: 18–23. doi:10.1016/j.anireprosci.2016.08.004

44. Garner JB, Chamberlain AJ, Vander Jagt C, Nguyen TTT, Mason BA, Marett LC, et al. Gene expression of the heat stress response in bovine peripheral white blood cells and milk somatic cells in vivo. Sci Rep. 2020;10: 19181. doi:10.1038/s41598-020-75438-2

45. Attia YA, El-Hamid AEA, El-Hanoun AM, Al-Harthi MA, Abdel-Rahman GM, Abdella MM. Responses of the Fertility, Semen Quality, Blood Constituents, Immunity and Antioxidant Status of Rabbit Bucks to Type and Magnetizing of Water. Ann Anim Sci. 2015;15: 387–407. doi:10.2478/aoas-2014-0086

46. Surai PF, Kochish II. Nutritional modulation of the antioxidant capacities in poultry: the case of selenium. Poult Sci. 2019;98: 4231–4239. doi:10.3382/ps/pey406

47. Park JH, Lee SI, Kim IH. Effect of dietary Spirulina (Arthrospira) platensis on the growth

performance, antioxidant enzyme activity, nutrient digestibility, cecal microflora, excreta noxious gas emission, and breast meat quality of broiler chickens. Poult Sci. 2018;97: 2451–2459. doi:10.3382/ps/pey093

48. Abdel-Moneim A-ME, Shehata AM, Mohamed NG, Elbaz AM, Ibrahim NS. Synergistic effect of Spirulina platensis and selenium nanoparticles on growth performance, serum metabolites, immune responses, and antioxidant capacity of heat-stressed broiler chickens. Biol Trace Elem Res. 2022;200: 768–779. doi:10.1007/s12011-021-02662-w

49. Khattab AAA, El Basuini MFM, El-Ratel IT, Fouda SF. Dietary probiotics as a strategy for improving growth performance, intestinal efficacy, immunity, and antioxidant capacity of white Pekin ducks fed with different levels of CP. Poult Sci. 2020; 100898. doi:https://doi.org/10.1016/j.psj.2020.11.067

50. Abd El-Hack ME, Alagawany M, Abdelnour S. Responses of growing rabbits to supplementing diet with a mixture of black and red pepper oils as a natural growth promoter. J Anim Physiol Anim Nutr (Berl). 2019;103: 509–517. doi:10.1111/jpn.13045

51. Abdelnour S, Hassan M, Mohammed A, Alhimaidi A, Al-Gabri N, Al-Khaldi K, et al. The Effect of Adding Different Levels of Curcumin and Its Nanoparticles to Extender on Post-Thaw Quality of Cryopreserved Rabbit Sperm. Animals. 2020;10: 1508. doi:10.3390/ani10091508

52. El-Bab AFF, Saghir SAM, El-Naser IAA, El-Kheir SMMA, Abdel-Kader MF, Alruhaimi RS, et al. The Effect of Dietary Saccharomyces cerevisiae on Growth Performance, Oxidative Status, and Immune Response of Sea Bream (Sparus aurata). Life. 2022;12: 1013. doi:10.3390/life12071013

53. Naga Raja Kumari K, Narendra Nath D. Ameliorative measures to counter heat stress in

poultry. Worlds Poult Sci J. 2018;74: 117–130. doi:10.1017/S0043933917001003

54. Gershwin ME, Belay A, editors. Spirulina in Human Nutrition and Health. CRC Press; 2007. doi:10.1201/9781420052572

55. Mirzaie S, Zirak-Khattab F, Hosseini SA, Donyaei-Darian H. Effects of dietary Spirulina on antioxidant status, lipid profile, immune response and performance characteristics of broiler chickens reared under high ambient temperature. Asian-Australasian J Anim Sci. 2018;31: 556–563. doi:10.5713/ajas.17.0483

56. Hamza RZ, EL- Megharbel SM, Altalhi T, Gobouri AA, Alrogi AA. Hypolipidemic and hepatoprotective synergistic effects of selenium nanoparticles and vitamin. E against acrylamide- induced hepatic alterations in male albino mice. Appl Organomet Chem. 2020;34. doi:10.1002/aoc.5458

57. Deng R, Chow T-J. Hypolipidemic, antioxidant, and antiinflammatory activities of microalgae Spirulina. Cardiovasc Ther. 2010;28: e33-45. doi:10.1111/j.1755-5922.2010.00200.x

58. Calogero AE, Condorelli RA, Russo GI, La Vignera S. Conservative Nonhormonal Options for the Treatment of Male Infertility: Antibiotics, Anti-Inflammatory Drugs, and Antioxidants. Biomed Res Int. 2017;2017: 1–17. doi:10.1155/2017/4650182
