# Supplementary material for: The synergistic impact of Spirulina and selenium nanoparticles mitigates the adverse effects of heat stress on the physiology of rabbits bucks
Source: PLoS One. 2023 Jul 12;18(7):e0287644. doi: 10.1371/journal.pone.0287644 (PMC10337889; doi:10.1371/journal.pone.0287644)
Supplement: Attachment — Submitted filename: PLoS One Comments.docx [file pone.0287644.s003.docx]

Comments

Title needs modification.

It’s not clear what the authors wanted study;

a) Does they wanted to study effect of spirulina on HS and nano Selenium on HS alone or in combination?

b) If authors wish to study the interaction then why there is not combination supplementation in the positive control group (non-heat stress exposed group).

c) How the combination will be better than the individual ones?

The experimental design is not correct.

*Introduction*

The authors failed to highlight the need for study or impact of heat stress on the semen/reproductive performance in bucks.

Authors further failed to explain why spirulina and nano Se has been selected for the study? As there is various polyphenolic compounds and nano forms of other minerals (Zn, Cr); why these nutrients or additives has been not used in the study?

Why combination study is needed?

Materials and methods

What is the selenium content in the CPD?

What is the NRC recommended level of Selenium in rabbit diets?

What is the duration of supplementation or experiment period?

How semen physical quality was assessed? Was manual method or CASA was used?

The authors did not presented/exhibited the heat stress in bucks by exposing to cyclic HS.

No information about feed consumption since, the HS first affects the intake.

No data on feed consumption, body weight change or water intake as indicator of HS induction in bucks.

The results seems contradictors as HS could results in haemo-concentration then the blood parameters should be increased in negative control group compared to the TNZ or positive control. No discussion about the blood parameters were presented.

Why post-thaw semen quality was assessed? How HS is correlated with post-thaw semen quality?

Discussion is not clear and supplementing the findings. Needs major improvement.

The authors failed to explain the synergism between the spirulina and Se.
