# Supplementary material for: The synergistic impact of Spirulina and selenium nanoparticles mitigates the adverse effects of heat stress on the physiology of rabbits bucks
Source: PLoS One. 2023 Jul 12;18(7):e0287644. doi: 10.1371/journal.pone.0287644 (PMC10337889; doi:10.1371/journal.pone.0287644)
Supplement: Attachment — Submitted filename: Comments on the manuscript.docx [file pone.0287644.s004.docx]

**Comments to the author(s):**

**Manuscript title:** The Synergistic Impact of Spirulina and Selenium Nanoparticles Mitigates the Adverse Effects of Heat Stress on Rabbits Bucks

The manuscript addresses a novel and vital topic for researchers, breeders, and others interested in the field. However, the following comments, inquiries, and suggestions may help improve the manuscript's quality.

1- How do you judge that the conditions in the positive group are thermal-neutral?
2- The hypothesis is not clear in the introduction. (See the comment (P:2) of the comments L:82-83).
3- I recommend calculating the change caused by the combination of Spirulina (SP) and two levels of selenium nanoparticles (SP-SeNPs) in a new table.

- Values that have already exceeded the average value = SP value + SP-SeNPs value/2 are placed. Keeping in mind that only the superior values are placed in the new table, and the values that are placed have a positive meaning, regardless of whether the values are + or -.
- Reference should be made to the new table which you will do with the results and discussion to clarify the meaning of the synergy between the combination of SP and SP-SeNPs.
- Indicate which of the two levels is the best or both according to the results in the new table and refer to this in the abstract and in the conclusion
- To obtain the change % in the following

1-	Calculating the average value of the (SP + SP-SeNPs).

2-	Calculating the value of the change in the value when the combination of SP and SP-SeNPs in both the two levels;

The change = recorded value - (1).

3-	Calculate the change as a percentage;

Change % = (2) / (1) X 100.

**Table 8. The percentage change in both levels due to the synergistic impact of the SP and SP-SeNPs.**

| **Item**s | **Average** | **Change %**  **SP+SeNPs25** | **Change %**  **SP+SeNPs50** |
| --- | --- | --- | --- |
| **Hematological variables** | | | |
| Hemoglobin | (11.93+ 10.5) /2  = 11.215 | (12.19-11.215) /11.215*100  = 8.69 | (12.28-11.215) /11.215*100  = 9.5 |
| RBCs | 6.18 | 2.75 | 2.91 |
| …ect |  |  |  |
| **Blood metabolites** | | | |
| …..ect |  |  |  |
| **antioxidant and oxidative marks in the blood and seminal plasma** | | | |
| **…. Ect** |  |  |  |
| **Semen production parameters** | | | |
| **…..** |  |  |  |
| **Sperm variables in post-thawed** | | | |
| **…. Ect** |  |  |  |
| **Blood and seminal testosterone** | | | |
| …..ect |  |  |  |
| **Reproductive performance of rabbits does** | | | |
| ……ect |  |  |  |

**In addition to:-**

**Line:28, 112** ……in the 1st group

Comment: a superscript letter

**Line 57-58:…..**

Comment: It is necessary to specify the type of change that occurred, whether by increase or decrease, rather than writing a change in general.

**Line 59, 328, 369.**

Comment: Put the whole term in the first time in L: 59, and the abbreviation in the next. The term ROS = reactive oxygen species.

**Line 63**………..and	antiparasitic	activities	and	contains

Comment: Remove the spaces.

**Line 67**……….. animal nuitrition, and the most

Comment: animal nutrition and the most.

**Line 70** . So, elements in NPs form…

Comment: So, elements in the NPs form..

**Line 73-74**…, metabolic processes, and is an essential trace element for animals.

Comment:….., and metabolic processes, and is an essential trace element for animals.

Line 74….Se is a crucial component in …….

Comment:… The Se is a crucial component in

**Line 82-83**: SeNPs and/or Spirulina (SP) were incorporated to mitigate the negative impacts on the heat-stressed growing rabbit by improving the antioxidant status.

Comment: Rewrite this sentence to be the research hypothesis.

**Line 97**………and 3.15±0.32 kg LBW

Comment: don't use the abbreviation (LBW) and use the full term "Live body weight".

**Line 97-99**…..Each buck served as a replicate and was kept individually in stainless steel cage batteries (40×50×35cm) accommodated with feeders and automatic drinkers in a closed system controlled by heat, humidity, and light regimes.

Comment: Please remove the spaces in the previous sentence.

**Line 113** … under normal indoor conditions.

Comment : write the (…-… °C; …-…RH%).

**Line 116 - 117**….

Comment : What was the basis for selecting the number of hours and days that you relied on in the negative control group?

**Line 125**…. The mean PDI was 0.13,

Comment : Don't use the abbreviation (PDI) and use the full term Polydispersity Index.

**Line 143**…. by using a phase-contrast microscope with a hot stage at 37°C

Comment: Remove the spaces.

**Line 161**

Comment: Tab.

**Line 176** ….. ear-vein of the does

Comment: ear-vein of the dose

**Line 268: Table 3**.. .. and their combinations on oxidative bio-marks in blood and...

Comment:….., and their combinations on antioxidant and oxidative marks in the blood and ….
