# Supplementary material for: The synergistic impact of Spirulina and selenium nanoparticles mitigates the adverse effects of heat stress on the physiology of rabbits bucks
Source: PLoS One. 2023 Jul 12;18(7):e0287644. doi: 10.1371/journal.pone.0287644 (PMC10337889; doi:10.1371/journal.pone.0287644)
Supplement: Attachment — Submitted filename: Comments of the revised manuscript.docx [file pone.0287644.s007.docx]

**Comments of the revised manuscript**

Manuscript title: The Synergistic Impact of Spirulina and Selenium Nanoparticles Mitigates the Adverse Effects of Heat Stress on Rabbits Bucks

All of my suggestions were incorporated into the revised manuscript.
