# Supplementary material for: The synergistic impact of Spirulina and selenium nanoparticles mitigates the adverse effects of heat stress on the physiology of rabbits bucks
Source: PLoS One. 2023 Jul 12;18(7):e0287644. doi: 10.1371/journal.pone.0287644 (PMC10337889; doi:10.1371/journal.pone.0287644)
Supplement: Attachment — Submitted filename: Response to Reviewers.docx [file pone.0287644.s008.docx]

**Responses to the comments of the Editor**

I found your manuscript interesting and fit the journal's scope. It has also improved well after the second revision round, but there are still some revisions that need to be fixed before accepting this paper.

Please make the modifications requested by the reviewers shown below (Reviewers, 2 and 6)

**Response:**

Thank you for your time and consideration. We have now changed this manuscript and inserted the required modifications with point-by-point responses to each of the reviewer’s comments. The corrected sentences were followed using **Track Changes** in the revised manuscript.

**Responses to the comments of Reviewer #2**

All comments have been addressed. Please look the attached file

**#Attached file:**

delete antioxidant and. oxidative markers consist of both antioxidants and pro oxidants (MDA)

**Response:** Thank you for your comment. Corrected in Table 3 as follows:

“**Oxidative markers in the blood”**

separate markers and in

**Response:** Thank you for your comment. Corrected in Table 3 as follows:

“**Oxidative markers in the blood”**

SAME AS ABOVE.

**Response:** Thank you for your comment. Corrected in Table 3 as follows:

“**Oxidative markers in the seminal plasma”**

**Responses to the comments of Reviewer #6**

The following corrections need to be made: Line 74….Se is a crucial component in …….Comment:… The Se is a crucial component in Response: Corrected as requested. Kindly check line 87. My comment: Above correction was not done.

**Response:** Thank you for your careful reading that ensures the perfection of the manuscript. Corrected in line 83 as follows:

**“**The Se is a crucial component in the…….”

Line 82-83: SeNPs and/or Spirulina (SP) were incorporated to mitigate the negative impacts on the heat-stressed growing rabbit by improving the antioxidant status. Comment: Rewrite this sentence to be the research hypothesis. Response: Done as requested. Kindly check lines 101-103.
My comment: L 98-99: The phrase “may affect the performance of male rabbits” is too general. Will the effect be positive or negative? What kind of performance in male rabbits will be affected?

**Response:** We greatly appreciate your constructive comment. Corrected in line 99 as follows:

“we hypothesize that a combination of SP with different levels of SeNPs may positively affect the reproductive performance of male rabbits under HS.”
