# Supplementary material for: The synergistic impact of Spirulina and selenium nanoparticles mitigates the adverse effects of heat stress on the physiology of rabbits bucks
Source: PLoS One. 2023 Jul 12;18(7):e0287644. doi: 10.1371/journal.pone.0287644 (PMC10337889; doi:10.1371/journal.pone.0287644)
Supplement: Attachment — Submitted filename: Response to Reviewers.docx [file pone.0287644.s010.docx]

**Responses to the comments of Editor**

**Comment:** Must be make the modifications requested by the reviewer 2. Please look the attached file and make all modified required.

**Response:** All comments have been addressed as suggested. Kindly check the revised version with track changes.

**Responses to the comments of Reviewer #2**

**Comment:** The authors did not address comments and review suggestions from my earlier review. I cannot suggest the publications of this manuscript in its current form.

**Response:** Thank you for your time and insightful comments. All comments have been addressed as suggested. Please check the revised version with track changes.

**#Attached file:**

Re-arrange your references. 2-5 cannot come before 1-2

**Response:** Done as suggested and citations start with No. 1. Please check line 54.

biomarkers

**Response:** Done as suggested. Kindly check Table 3.

delete antioxidant and. oxidative markers consist of both antioxidants and pro oxidants (MDA)

**Response:** Thank you for your comment. Corrected in Table 3 as follows:

“**Oxidative markers in the blood”**

separate markers and in

**Response:** Thank you for your comment. Corrected in Table 3 as follows:

“**Oxidative markers in the blood”**

SAME AS ABOVE.

**Response:** Thank you for your comment. Corrected in Table 3 as follows:

“**Oxidative markers in the seminal plasma”**

This caption in the title is not a true reflection of what was done. heamato-biochemical, semen quality, testosterone, oxidative status and sperm characteristics were all reported in this study. is suggest the use of physiology in place of semen quality in the title or list the above indices in the title, if not too lengthy

**Response:** Done as suggested as follows (Please check the title):

**The Synergistic Impact of Spirulina and Selenium Nanoparticles Mitigates the Adverse Effects of Heat Stress on the Physiology of Rabbits Bucks**

reference 2-5 cant start the manuscript. Re-number from 1.

**Response:** Done as suggested and citations start with No. 1. Please check line 54.

initial corrections pointed out in the earlier review are not addressed.

**Response:** Done as suggested. Kindly check Table 3.

oxidative markers are a combination of antioxidant and prooxidant indicators. hence oxidative markers will suffice.

**Response:** Done as suggested. Kindly check Table 3.
